# BioStructNet: Structure-Based Network with Transfer Learning for Predicting Biocatalyst Functions

**DOI:** 10.1021/acs.jctc.4c01391

**Published:** 2024-12-20

**Authors:** Xiangwen Wang, Jiahui Zhou, Jane Mueller, Derek Quinn, Alexandra Carvalho, Thomas S. Moody, Meilan Huang

**Affiliations:** † School of Chemistry and Chemical Engineering, Queen’s University Belfast, BT9 5AG Belfast, Northern Ireland, U.K.; ‡ Department of Biocatalysis and Isotope Chemistry, Almac Sciences, BT63 5QD Craigavon, Northern Ireland, U.K.; § Arran Chemical Company Limited, Unit 1 Monksland Industrial Estate, Athlone, Co. Roscommon N37 DN24, Ireland

## Abstract

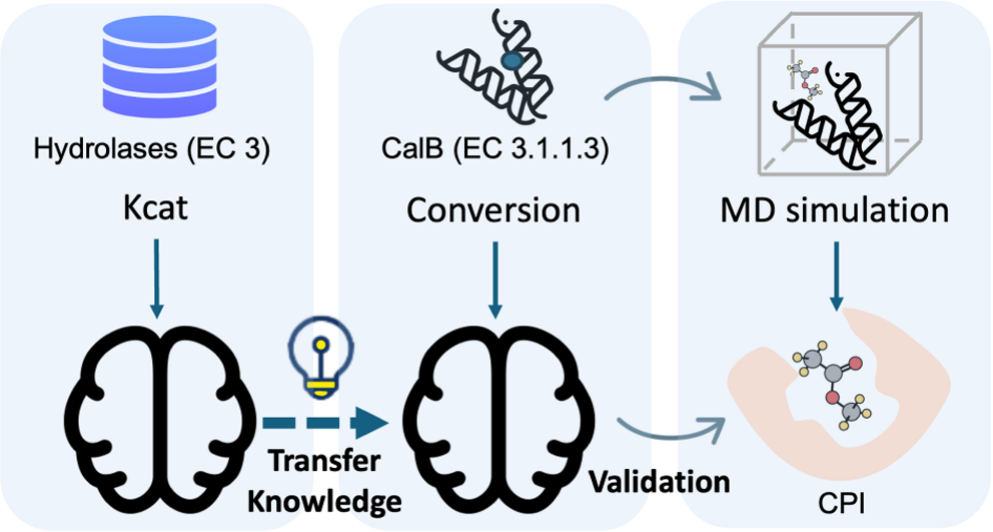

Enzyme–substrate interactions are essential to both biological processes and industrial applications. Advanced machine learning techniques have significantly accelerated biocatalysis research, revolutionizing the prediction of biocatalytic activities and facilitating the discovery of novel biocatalysts. However, the limited availability of data for specific enzyme functions, such as conversion efficiency and stereoselectivity, presents challenges for prediction accuracy. In this study, we developed BioStructNet, a structure-based deep learning network that integrates both protein and ligand structural data to capture the complexity of enzyme–substrate interactions. Benchmarking studies with different algorithms showed the enhanced predictive accuracy of BioStructNet. To further optimize the prediction accuracy for the small data set, we implemented transfer learning in the framework, training a source model on a large data set and fine-tuning it on a small, function-specific data set, using the CalB data set as a case study. The model performance was validated by comparing the attention heat maps generated by the BioStructNet interaction module with the enzyme–substrate interactions revealed from molecular dynamics simulations of enzyme–substrate complexes. BioStructNet would accelerate the discovery of functional enzymes for industrial use, particularly in cases where the training data sets for machine learning are small.

## Introduction

Compound–protein interactions (CPIs) play a crucial role in the design of enzymes as biocatalysts, and the accurate and fast prediction of CPIs can drive innovations in biocatalysis and related fields. Although experimental assays remain the most reliable approach for determining CPIs, the vast costs and labor required to experimentally characterize every potential protein–ligand pair make this approach prohibitive. In recent years, computational methods, particularly machine learning techniques, have greatly advanced the CPI prediction.^1,2^ Compared to the deep learning-based protein structure prediction, for which there are already established data sets, i.e., CASP,^3,4^ little has been reported for assessing the biocatalysis properties predicted by machine learning. The lack of standardized protocols and benchmark data sets, coupled with the discrepancies in the performance measurements, presents significant challenges in developing accurate predictive models in the enzyme catalysis arena.^5^ Moreover, most machine learning (ML) models showed limited predictive accuracy due to overfitting when applied to small, function-specific data sets, especially for protein sequences with high similarity. Consequently, there is an urgent need for innovative methodologies that can effectively leverage limited function-specific enzyme data by applying the insight gained from large data sets for improving the prediction accuracy for small data sets.

As powerful tools for CPI prediction, machine learning algorithms utilize large data sets to uncover complex patterns,^6^ using various techniques such as random forest (RF),^7^ support vector machine (SVM),^8^ Gaussian processes,^9^ and boosting.^10^ In recent years, deep learning algorithms such as convolutional neural networks (CNNs) and graph neural networks (GNNs) have proven effective in predicting enzyme functions based on the features retrieved from protein sequences.^11−20^ With the recent increase in protein structural data and protein–ligand complexes data sets, structure-based machine learning methods^21−24^ have been reported, where proteins are represented through sequential CNNs or two-dimensional (2D) pairwise distance maps.

Despite the emerging machine learning models for CPI prediction, these models have limited performance in predictive accuracy when applied to small, function-specific data sets due to overfitting and the lack of diverse training examples.^20^ This limitation requires the development of novel methods that can effectively leverage the available data while mitigating the impact of data set size. Transfer learning, which involves pretraining a model on a large data set and fine-tuning it on a smaller, task-specific data set, offers a promising solution.^25,26^ By transferring knowledge from a broad, general model to a specialized model, transfer learning can enhance the prediction accuracy for function-specific enzyme–substrate interactions.

Compared with machine learning methods, physics-based computational approaches such as molecular docking and molecular dynamics (MD) simulations are also used for CPI prediction. However, careful analysis is required when using these methods due to the challenges in conformational sampling. Deep learning was integrated with molecular docking results in drug discovery, where MD simulated trajectories were employed as the input features of machine learning models to improve the prediction accuracy^27,28^ or enhance the efficiency of traditional docking-based screening.^29,30^ Enlighted by these advances, we propose to concinnate the data-based parameters with physics-based computational simulations to validate and interpret the deep learning model’s predictions. This approach could be used to assess the reliability and accuracy of the deep learning predictors in the context of enzyme–substrate interactions.

One enzyme of particular interest for CPI prediction and application is *Candida antarctica* lipase B (CalB).^31^ In recent years, the rapid increase in plastic waste has highlighted the need for innovative solutions to degrade plastics efficiently. CalB has emerged as a promising biocatalyst due to its high thermostability and effectiveness in breaking down polyethylene terephthalate (PET) into terephthalic acid (TPA), a key step in plastic degradation.^32^ Moreover, CalB can degrade other plastics, such as ε-polycaprolactone, further underscoring its potential in environmental applications.^33^ CalB belongs to the EC3 hydrolase class, which hydrolyzes the ester bond in a large and promiscuous binding pocket with an oxyanion hole, representing a challenging case for structure-based machine learning. The growing importance of CalB in plastic degradation and its challenging structural and mechanism features make it an ideal case study for developing and validating a robust CPI prediction model, especially for small and function-specific data sets.

In this study, we developed a novel structure-based deep learning model BioStructNet to address the challenges of small, function-specific enzyme data sets, using CalB as a case study. The CalB database contains a limited number of sequences derived from the wild-type protein, therefore presenting a challenging case for developing machine learning models for CPI prediction. To address this challenge, we developed BioStructNet, utilizing transfer learning to transfer the knowledge learned from larger data sets, to enhance the model’s ability to generalize in small data sets like CalB. Protein graphs are generated based on residue coordinates and assign physicochemical property features assigned to nodes, while ligand graphs are created using SMILES. These structure-based graph representations are combined in the interaction module to generate contact heat maps for specific protein–ligand pairs, which are then used for regression or classification predictions. By combining structural representations with transfer learning, BioStructNet significantly improves the prediction accuracy for CalB and similar small data sets. The high-scoring residues predicted by the deep learning model’s attention weights are in agreement with the key protein residue sites identified through docking and MD simulations, which validated the model’s reliability. Our BioStructNet transfer learning approach provides a promising solution to improve CPI prediction accuracy for small, specific data sets and would accelerate the discovery of effective biocatalysts for specific functions.

## Results and Discussion

### BioStructNet Framework

**Figure 1:**
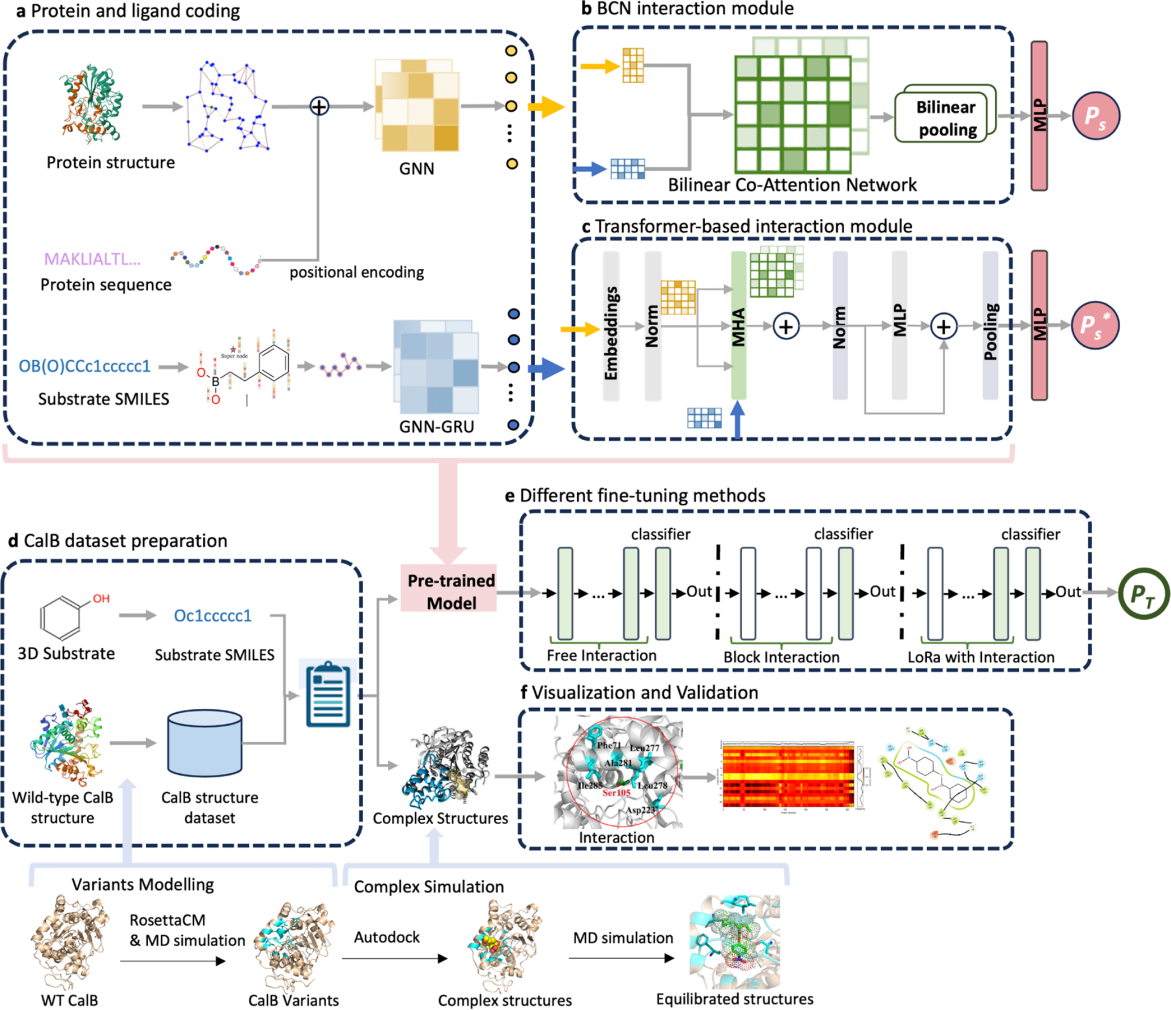
BioStructNet framework with two sections: processing the source task data set (a–c) and transfer learning and validation (d–f). (a) Embeddings of Proteins and Ligands. Proteins and ligands are encoded by GNNs, with substrate structures encoded by SMILES and protein structures denoted by three-dimensional (3D) contact maps and sequence-based positional embeddings, to preserve spatial relationships. (b) BCN Interaction Module. The Bilinear Co-Attention Network utilizes two fully connected networks to linearly transform the input features of proteins and ligands, followed by a bilinear multiplication to compute the attention weights. (c) Transformer-Based Interaction Module. This module with multihead self-attention on embedded protein features preceding a cross-attention with ligand features, is embedded with layer normalization and implemented in a feed-forward network (FFN). (d) CalB Data Preparation. Protein structures of CalB variants were generated by RosettaCM and refined by MD simulations. Autodock is used to dock the CalB variants with ligands to give complex structures, which are then analyzed by MD simulations to evaluate interactions. (e) Different fine-tuning strategies, including free interaction, block interaction, and LoRa for model adaptation. (f) Visualization of protein–ligand interactions and attention weights predicted by BioStructNet.

CalB belongs to the hydrolase enzyme family, which hydrolyzes ester substrates through reactions with water. Despite its significant industrial importance, the limited availability of data makes it difficult to build accurate deep learning models for CalB. To address this challenge, we designed a BioStructNet framework (Figure 1) to predict the function of small target data sets by leveraging the knowledge learned from the large source data sets.

The BioStructNet framework is divided into two main sections: modules for processing the source task data set (blocks a, b, and c); and modules for transfer learning and validation (blocks d, e, and f). Protein structures are encoded using contact map graphs, which are comprised of edges represented by the spatial distance between the α carbons of two residues within a defined distance cutoff and node features assigned with residue properties.^34^ Simultaneously, ligand structures are encoded by graphs comprised of edges and nodes represented by atomic bonds and atom types (Figure 1a). Attention mechanisms are employed in two independent interaction models to model CPIs: the bilinear coattention network (BCN), which captures short-range CPI information, and the transformer-based interaction module, which integrates global protein features to capture long-range CPIs by applying multihead attention (MHA) among protein amino acids (Figure 1b,1c). The transformer-based interaction module also includes layer normalization and a feed-forward network, which enhance the stability and expressiveness of the model by normalizing intermediate representations and applying nonlinear transformations, respectively. The transfer learning module employs three fine-tuning methods: a free interaction module, where all layers interact freely, allowing flexibility in adapting to new data; a block interaction module, where interaction layers are selectively constrained to preserve the structure of the pretrained model; and a LoRa fine-tuning method, which combines low-rank adaptation (LoRa) with structured interactions to balance model efficiency and flexibility (Figure 1e). The training process utilizes 5-fold cross-validation and bootstrapping to ensure prediction accuracy.

The source model is built on a source database of hydrolases with turnover number (*K*_cat_). The model performance for regression tasks is evaluated on its predictive accuracy for enzyme activity for substrates reflected by enzymes’ catalytic efficiency. Additionally, the predictive capability of the model for classification tasks is demonstrated using a widely applied human compound-protein interaction (CPI) data set, which allows the differentiation of enzymes with distinct activity level reflecting different enzyme–substrate binding affinities. The target model is built on a CalB conversion database. The protein structures of CalB variants generated by comparative modeling using RosettaCM and refined by molecular dynamics (MD) simulations, serve as the input for the transfer learning. For model validation, the complex structures obtained by docking followed by MD simulations were inspected to assess the protein–ligand interactions in relation to the attention weights of the model.

### BioStructNet Performance on the Source Data Sets

The performance of BioStructNet was evaluated on regression task using the *K*_cat_ “EC 3” (hydrolase) data set and classification tasks with Human CPI data set, in comparison to various baseline models, including random forest (RF), *k*-nearest neighbors (KNN), L2, Tsubaki’s,^35^ DLKcat,^19^ DrugVQA,^21^ TransformerCPI2.0,^36^ ALDELE^20^ and DrugBAN^16^ (Table 1). For the regression task, root mean square error (RMSE) and *R*^2^ were used as the metrics of model performance, while for the classification task, area under the curve (AUC), recall, and precision were used.

**Table 1:** Benchmark Studies of the Performance of Machine Learning Models in Regression and Classification Tasks, Using the *K*_cat_ “EC 3” Data Set (for Regression Task) and the Human Data Set (for Classification Task), Respectively

data set	collective *K*_cat_		human		
models	RMSE	*R*^2^	AUC	recall	precision
RF	4.08	0.37	0.940	0.897	0.861
KNN	4.49	0.24	0.860	0.927	0.798
L2	4.63	0.03	0.911	0.913	0.861
Tsubaki’s^a^			0.970	0.918	0.923
DLKcat^a^	5.41	0.30			
DrugVQA	4.13	0.03	0.964	0.948	0.897
TransformerCPI	5.85	0.17	0.973	0.925	0.916
ALDELE	4.95	0.40	0.974	0.929	0.904
DrugBAN	5.18	0.01	0.924	0.939	0.921
BioStructNet-BCN	3.11	0.35	0.930	0.931	0.927
BioStructNet-Transformer	2.99	0.37	0.898	0.890	0.876

a Tsublk’s and DLKcat have similar structures as their respective baselines, with output for classification task and regression task, respectively.

In the regression task (*K*_cat_ data set), a 5-fold cross-validation was performed by splitting the data into training, validation, and test sets with a ratio of 7:1.5:1.5. The final model was selected based on the best *R*^2^ score on the validation set, and performance metrics were averaged across the 5-fold to ensure robustness. Our BioStructNet model, with both BCN and Transformer interaction modules, achieved better RMSE (2.99 and 3.11) and *R*^2^ scores compared with baseline models, indicating improved performance in predicting *K*_cat_ values. Additionally, we evaluated BioStructNet on the BindingDB *K*_d_ binding affinity data set which represents a large-scale CPI data set (with more than 50k entries), to assess the model’s prediction performance for regression tasks and excellent predictive capabilities were also attained. The data set cleansing process and BioStructNet’s performance with the curated BindingDB data set are presented in Supporting Figure S1.

We also benchmarked our BioStructNet with other approaches for handling classification tasks (predicting interaction or noninteraction, represented as 0 or 1), using the widely used human CPI data set. Among all, the sequence-based models performed particularly well (AUC around 0.97), such as ALDELE, TransformerCPI, and Tsubaki’s. These models are exclusively based on sequence information; hence, they can be utilized to train larger data sets and demonstrate obvious advantages in terms of data coverage, as reflected in their high AUC, recall, and precision scores. It is important to note that retraining sequence-based models on a smaller structure-based data set would not only diminish their inherent advantage in data scalability but would also lead to an unfair evaluation scenario. Sequence-based models are specifically designed to leverage large data sets, which is a core aspect of their strength. To ensure fairness, we evaluated our BioStructNet models, particularly the BCN interaction module, on the structure-based data set. These models showed competitive precision and recall scores, even when compared to sequence-based models trained on a larger data set. This demonstrates the robustness of BioStructNet in handling structural information, highlighting its potential for specific biocatalysis applications, particularly in scenarios in which predictive accuracy and minimizing false positives are critical.

**Figure 2:**
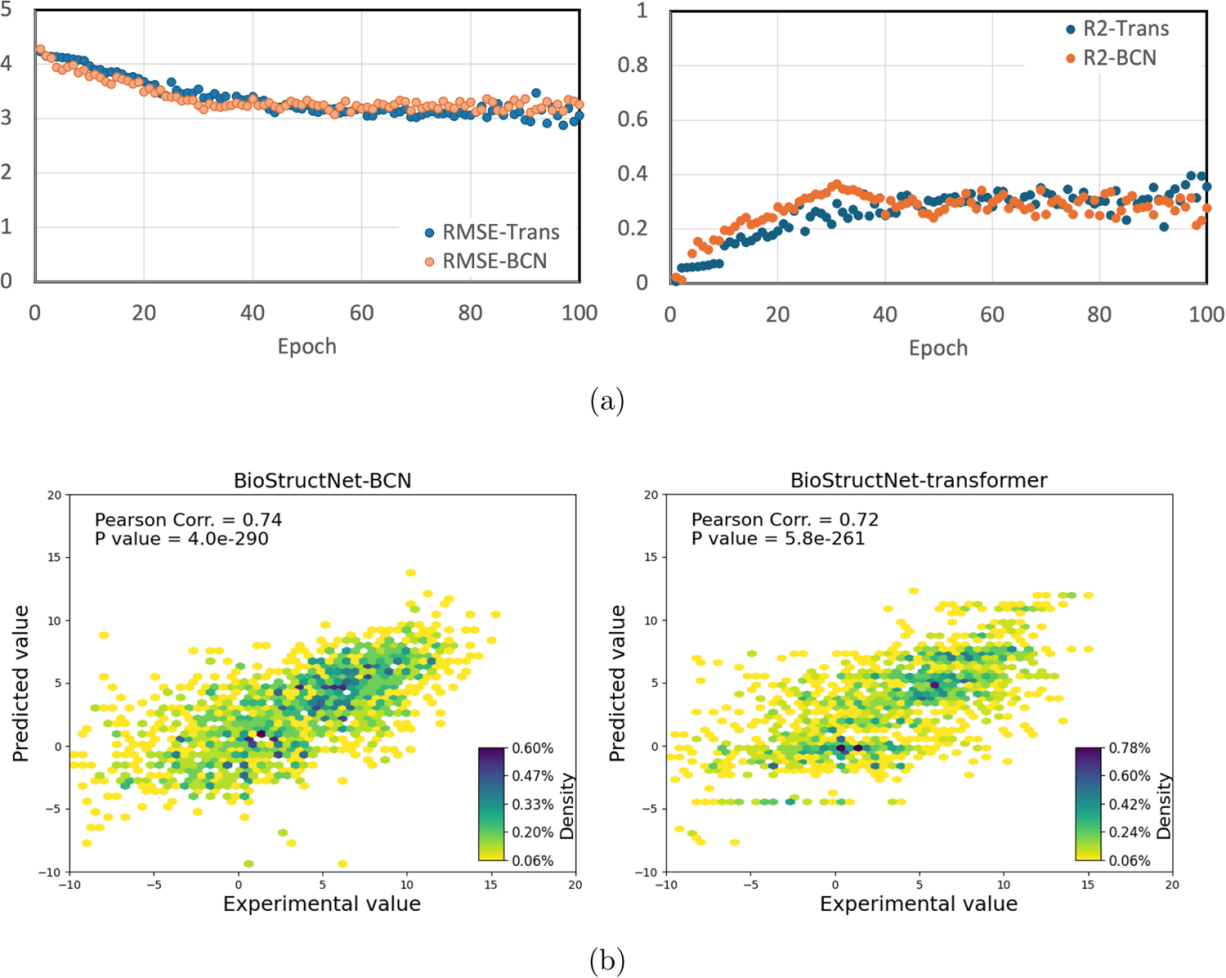
Performance of BioStructNet models for *K*_cat_ prediction with the BCN interaction module and transformer-based interaction module. (a) The RMSE and *R*^2^ of the validation set during the training process. (b) Performance of the final deep learning models. The correlation between predicted *K*_cat_ values and the experimental values in the whole data set (training, validation, and test data sets) was evaluated. The brightness of color represents the density of data points. *P* values for Pearson’s correlation were calculated.

The BioStructNet models trained with the *K*_cat_ “EC 3” data set for the source task serve as the foundation for the CalB task with the performance shown in Figure 2. The RMSE and *R*^2^ values for the validation set during the training process are shown in Figure 2a, and the performance of the final BioStructNet models—both with the BCN interaction module and the Transformer-based interaction module are shown in Figure 2b. The BioStructNet-BCN model showed an RMSE of 3.11 and an *R*^2^ of 0.35. The BioStructNet-Transformer model showed a slight advantage over the BCN module with an RMSE of 2.99 and an *R*^2^ of 0.37. Because it implements a self-attention for protein information prior to the cross-attention with ligand, it is able to effectively capture global contexts and long-range dependencies in protein–ligand interactions. Therefore, to improve prediction accuracy and generalization, it is necessary to accurately represent complex interactions, by capturing both local and global interaction patterns using structural features encoded in a graph neural network.

### BiostructNet on the Target Data Set

The BiostructNet models are trained on a CalB conversion data set with variants derived from the wild-type enzyme. The performance of the models was evaluated on the target CalB data set for both regression and classification tasks, with a focus on implementing transfer learning in classification to improve prediction accuracy for small data sets.

First, the model was trained for the regression task, and the performance is shown in Table 2. As expected, the results are inferior to those trained based on the large hydrolase *K*_cat_ data set (Table 1). Among the tested models, BioStructNet-BCN exhibited the best performance with an RMSE of 9.31 and an *R*^2^ of 0.37, while the BioStructNet-Transformer performed moderately with an RMSE of 12.18 and an *R*^2^ of 0.09. Unlike the models trained on the large *K*_cat_ data set, where the BioStructNet-Transformer interaction module performed better, the BCN interaction module shows better performance on the small CalB conversion data set. The CalB data set is derived from the same wild-type protein and contains mutations around a few key positions, such that they all share similar structures and likely play a crucial role in determining the conversion rates. Unlike the large hydrolase *K*_cat_ data set, which benefits from capturing long-range structural interactions due to the diversity of its enzyme–substrate pairs, the CalB data set is dominated by local structural changes. Consequently, the bilinear attention mechanism effectively captures these subtle local differences, resulting in superior performance compared with the global self-attention of the Transformer module. Enhancing the diversity of proteins in the CalB data set could potentially improve the model’s performance. Tailored sampling strategies or a weighted loss function could help mitigate the impact of rare cases and reduce prediction errors at the distribution’s tails. Furthermore, further expanding the data set to include a broader range of mutations, especially those with diverse structural features, could reduce data imbalance and improve the model’s generalizability.

**Table 2:** Performance Comparison on the CalB Conversion Data Set for the Regression Task

models	RMSE	*R*^2^
RF	16.45	–0.17
KNN	15.67	–0.01
DLKcat	13.68	0.13
TransformerCPI	13.96	0.10
ALDELE	11.11	0.32
BioStructNet-BCN	9.31	0.37
BioStructNet-Transformer	12.18	0.09

In view of the deficient performance shown in the regression task for the CalB data set, a classification approach was explored to improve the prediction accuracy. A binary classification approach with multiple thresholds was evaluated. Three fine-tuning methods, free, block, and LoRa, were systematically applied to the BCN interaction module. The transfer learning performance on the CalB conversion data set is evaluated by AUC, accuracy, relative error (RE), as well as precision and recall score, using three different classification thresholds: 15, 30, and 40% (Table 3). The 15% cutoff was applied to distinguish CalB variants with low conversion rates from moderate or high rates, to rule out the least active enzymes from the subsequent experimental validations. The 30% cutoff serves as an intermediate threshold, marking the variants with moderate enzyme activities, while the 40% cutoff distinguishes the variants with relatively high conversion rates, labeling the enzymes with exceptionally high conversion. The precision and recall scores are particularly low. This is likely due to the small size of the CalB data set, which limits the model’s ability to learn robust and generalizable features and capture subtle class distinctions and hence leads to more false positives and false negatives. The performance of the BioStructNet transfer model is averaged across 100 bootstrapping iterations. The fine-tuning results for the transformer-based module for the source data set are provided in Supporting Table S1.

**Table 3:** Performance of Transfer Learning Models Fine-Tuned on the BCN Interaction Module was Evaluated on the CalB Conversion Data Set for the Classification Task

cutoff	models	AUC	accuracy (%)	1.0% RE	precision	recall
15	Free BCN	0.63	81.38	0.28	0.58	0.56
	Block BCN	0.71	82.47	0.30	0.65	0.59
	LoRa BCN	0.71	83.41	0.31	0.65	0.60
30	Free BCN	0.64	66.27	0.45	0.59	0.57
	Block BCN	0.68	68.85	0.43	0.69	0.65
	LoRa BCN	0.69	72.71	0.47	0.69	0.67
40	Free BCN	0.66	62.62	0.48	0.61	0.60
	Block BCN	0.69	65.76	0.44	0.68	0.67
	LoRa BCN	0.66	68.17	0.48	0.69	0.67

The results indicate that the LoRa BCN model consistently outperforms the other methods across all thresholds. At the 15% cutoff, LoRa BCN achieves the highest accuracy (83.41%) and a strong AUC (0.71), effectively distinguishing the CalB variants with low conversion rates from those with moderate and high rates. The accuracy of the fine-tuning model with a 30% cutoff and a 40% cutoff is inferior to that with the 15% threshold. This indicates the classification threshold would affect the model performance and for the CalB conversion data set, a low cutoff value would be valuable to exclude the nonfunctional variants and hence reduce the variant library size for subsequent experimental evaluation.

**Figure 3:**
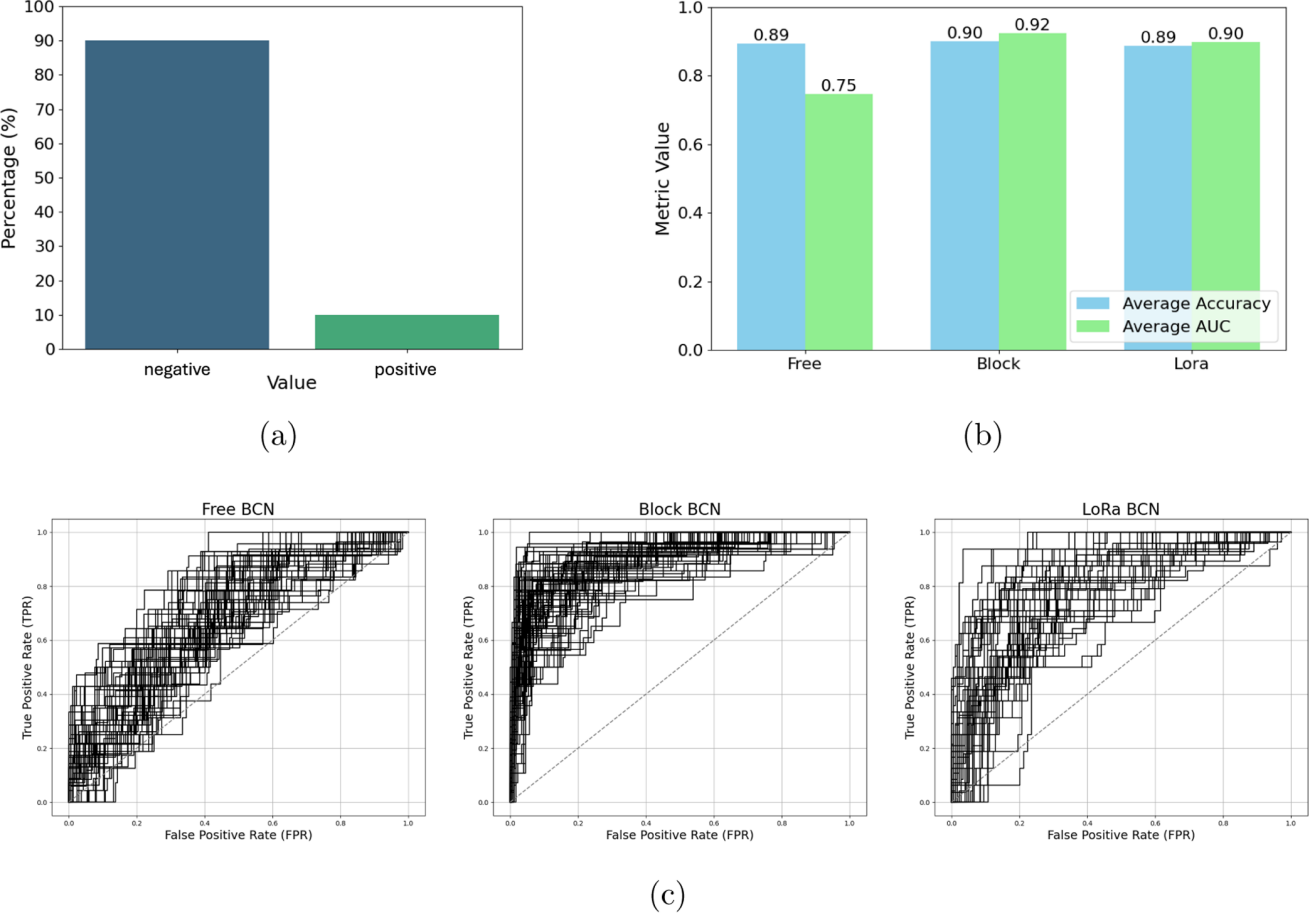
Performance of BioStructNet-BCN transfer learning models on the CalB enzyme data set, which was classified based on the relative activity of the variants in comparison with the wild-type and an augmentation approach. (a) Percentage distribution (0 and 1) of the augmented data set. Approximately 90% of the values are 0, while around 10% of the values are 1. (b) Comparison of average accuracy and AUC for the three fine-tuning strategies (Free, Block, and LoRa). (c) The receiving operating characteristic (ROC) curves for the final-epoch results of each boosting iteration, show the false positive rate (FPR) and true positive rate (TPR) across the three fine-tuning methods: Free, Block, and LoRA. Each solid black line represents the ROC for a boosting iteration, while the dashed black line shows the random classification.

The CalB data set includes the conversion data of wild-type and variant proteins for various substrates. In addition to the above classification with different thresholds, numerical binary classification is made based on the relative conversion value of a mutant’s activity compared with that of the wild-type protein for the same substrate: i.e., if the mutant performs comparably or better (taking into consideration of the experimental errors), it is classified as 1; otherwise, it is classified as 0. This method helps identify functional differences and informs beneficial mutation engineering. The CalB data set is small with mutation positions populated only around several positions. Such small data sets can lead to performance variability. To improve the model’s robustness, we implemented a data augmentation strategy grounded in domain-specific knowledge of CalB enzyme catalysis. Considering mutations near active site residues around the catalytic triad Asp187, Ser105, and His224 (first and second sphere regions)^37^ are more likely to affect the enzyme function than the mutations farther away, the large number of mutations outside the binding site reflects the proportionate difference in size between the outside binding regions and the binding site. 1208 single-point mutation variants, labeled as negative samples, were generated that covered all the residues (151) located far from the catalytic core (20 Å beyond the catalytic triad); and 8 random single-point mutations were performed for each position. As a result, the CalB database consists of 145 positive and 1208 negative samples (Figure 3a), which reflects the coverage of structural features outside the active site and ensures the diversity in mutation types which enhances the model’s ability to generalize during machine learning training.

The comparison of model performance metrics, accuracy, and AUC (Figure 3b) and the receiving operating characteristic (ROC) curves across three fine-tuning methods, Free, Block, and LoRa shows the trade-offs between parameter flexibility and generalization in transfer learning (Figure 3c). The average AUC and accuracy for all methods surpass models with different conversion thresholds, with an accuracy of 90% and AUC of 0.90, indicating the superior performance of the binary classification compared to the classification with definite thresholds (Table 3).

The BioStructNet with transfer learning achieved good performance for predicting the conversion of CalB regardless of the classification approaches, either categorical or numerical binary, effectively transferring the enzyme–substrate interaction information learned from the *K*_cat_ source data set to the CalB conversion prediction. Due to the high sequence similarity in the target data set, the diversity among different samples in the data set are minimal, limiting the model’s ability to learn sufficient diversity. Therefore, leveraging the broad enzyme–substrate interaction information from the source *K*_cat_ data set, which encompasses the diversity and complexity of protein binding with various small molecules.

Free fine-tuning, which adjusts all interaction parameters freely, offers greater flexibility during parameter optimization; however, it would result in noise and overfitting particularly for the small target data set, as shown by the scattered ROC results and fluctuations at low FPR (Figure 3). In contrast, Block fine-tuning, which constrains the majority of the parameters in the original network, only adjusting the classifier and the final layer of the network during optimization, retaining interaction information from the *K*_cat_ source data set, and hence achieving high accuracy with a steep ROC curve. LoRa fine-tuning employs low-rank adjustments to fine-tune the local network, retaining the performance of the pretrained model on the source data set while adapting for the new target data set by minimizing the parameter adjustment, as demonstrated by a stable ROC curve. Thus, it is crucial to select the appropriate fine-tuning strategy, and the choice of fine-tuning methods should be guided by the prediction tasks for a specific target data set.

Overall, the BioStructNet framework with transfer learning approaches, with its ability to capture local protein–ligand interaction patterns by the BCN interaction module, shows robust performance across different classification thresholds in the classification tasks for small function-based data set such as the CalB conversion data set.

### Model Validation and Attention Visualization

For enzyme engineering, it is critical to identify the amino acid residues and interaction pairs of compound-protein complexes responsible for the catalytic activities of enzymes. BioStructNet’s attention mechanism captures the importance of features of interactions between protein residues and ligand atoms in the CPIs. The atom pairs with higher attention weights denote stronger or more dominant CPIs ascribed to both spatial distances and the physiochemical properties of amino acids. The protein–ligand interactions concentrate on the spatially close interacting pairs, so the atom pairs in the first sphere of the catalytic site tend to have higher attention weights.

To validate the predicted CPIs responsible for the conversion disclosed by the machine learning models, we investigated the binding modes of wild-type and mutant CalB enzymes using docking and MD simulations. The experimental results are summarized in Supporting Table S2 to facilitate a direct comparison. The structures of the involved ligands are presented in Figure S2. Principal Component Analysis (PCA) of the equilibrated MD trajectories, as demonstrated by the root-mean-square deviation (RMSD) analysis (Supporting Figure S3), was performed to reduce the data into principal components (Supporting Figure S4). *K*-means clustering was applied to identify the dominant conformations of the protein–ligand complexes, which were then analyzed in relation to the attention weights from BioStructNet models (Supporting Figure S5).

**Figure 4:**
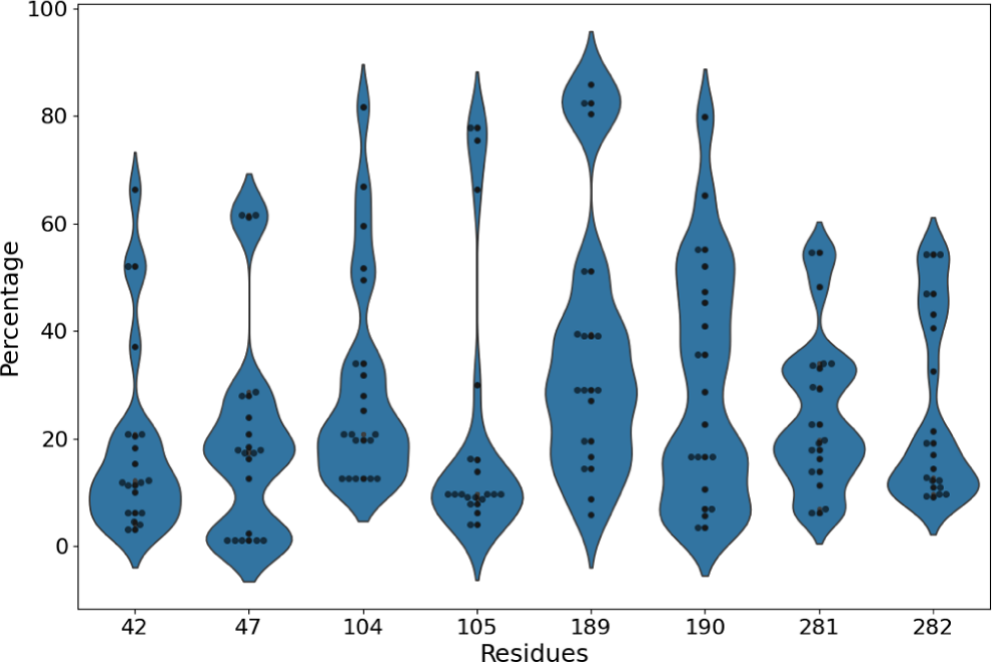
Violin plots of the attention weight distribution representing different protein residues of wild-type CalB interacting with 25 various ligands. Wider regions indicate higher attention density at specific residue positions. The top *n*% of residues with the highest attention weights are populated around the lower regions of the violin at certain positions, highlighting the most relevant positions for ligand interactions.

The distribution of attention weights for the CPIs between the wild-type enzyme with 25 various ligands is summarized in Figure 4 at the 8 specific residues experimentally selected for mutation engineering.^38^ Here, the “top percentages” represent the top *n*% of residues with the highest attention weights, indicating the most relevant positions identified for interactions with the ligands. For example, the attention weights are highly concentrated between 0 and 20% for residues T42, S105, and A282, indicating that these positions are critical for interactions with the 25 different ligands involved in the study. The attention weights for residues S47 and W104 are primarily concentrated around 20%, although slightly spanning to the top 40%, indicating diverse interaction potentials across different CPIs. Two hydrophobic residues I189 and V190 exhibit a wide range of attention weights, reflecting significant variability in the effect of mutations across different interactions. The attention weights for residue A281 are predominantly concentrated between 20 and 60%, with a noticeable peak at around 50%.

**Figure 5:**
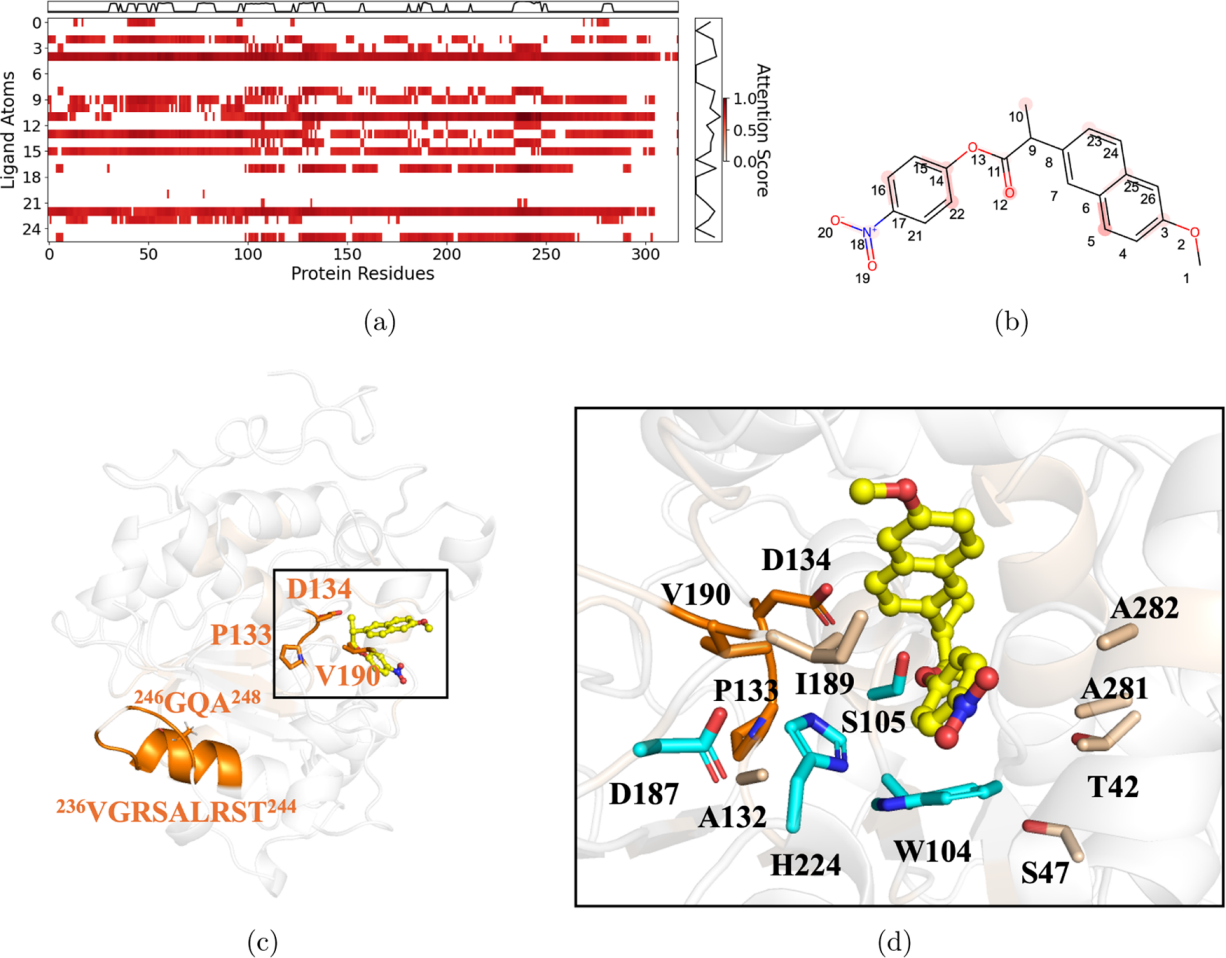
Explainability analysis of the wild-type CalB structure with ligand rac-10. (a) Attention heat maps for protein residues and ligand atoms, where darker color indicates higher attention weights. Only the points with the top 30% of attention weights are retained with their original values, with the others set to 0. Line plots above and on the right of the heat map show the average attention scores for residues and ligand atoms. (b) Structure of ligand rac-10 with attention weights. The ligand atoms with high attention scores (responsible for CPIs) are highlighted in red. (c) The three-dimensional interaction map of the wild-type CalB protein structure (PDB ID: 1TCA) and the ligand rac-10. The substrate is represented by a ball-and-stick model and marked in yellow. Residues with top 5% attention weights are highlighted in orange color. d. The binding pose of substrate rac-10. The catalytic triad Asp187, Ser105, His224, and the key residue W104 are marked in cyan. The active site residues that are ranked within the top 30% of attention weights are highlighted in pink, while those in the top 5% are marked in orange.

Here, the CPI between the wild-type protein and ligand rac-10 was shown as an example.^38^ The attention scores generated by BioStructNet were normalized to a 0–1 range to ensure consistency in the representations. The attention maps were averaged across all heads to obtain a single attention score matrix between ligand atoms and protein residues, with colors representing attention weights ranging from 0 (bright) to 1 (dark) (Figure 5a). The line plots above and on the right sides of the interaction map represent the mapping of attention scores along the direction of each protein residue or ligand atom, illustrating the distribution of attention across the nodes.

We then compared the attention weight map with the simulated structure of CalB in complex with rac-10 (Figure 5). The simulated complex was obtained by docking the ligand into the wild-type CalB protein (PDB ID: 1TCA) followed by MD simulations. For ligand rac-10, the carbon atoms on its benzene ring show notably high attention weights (Figure 5b), indicating that the interactions with the protein residues at these specific sites contribute largely to the overall CPI. This is consistent with the results observed in the MD simulation results, where W104 was found to form a π–π interaction with the phenyl ring of rac-10 (Figure 5d). Previous mutagenesis studies showed that mutations at W104 would affect the enzyme’s activity and enantioselectivity.^39^ Interestingly, two second sphere residue sites, T42 and S47 surrounding W104, are also associated with high attention weights. The attention weight importance observed for the third-sphere residues A281 and A282 may be attributed to their proximity to the second sphere residue T42. Notably, mutagenesis experiments have shown that the combined mutation at A281 and A282 enhanced *K*_cat_ and conversion for ligand rac-10.^38^ The role of I189 and V190 on CPIs is possibly attributed to their special proximity to another catalytic triad H224. In addition, residues 132–134 (Figure 5c,d) were also found to have high attention weights (ranked top 10%). This is possibly because of the hydrophobic interactions between A132 and key catalytic residue H224. These distantly located residues could affect the enzyme’s catalytic efficiency by indirectly reshaping the active site via the interactions with the first sphere residues. The good agreement between the attention modules in our BioStructNet models and the interaction patterns between protein residues and ligand atoms from the visual inspection indicate it may serve as a valuable tool to prioritize the important sites for mutation, and to improve the efficiency of enzyme engineering.

It should be noted that the residues with high attention weights affecting *K*_cat_ and conversion are not limited to the active site. In addition to those residues located in the proximity of the catalytic site, some sites with high attention weights in CPI predictions are far from the catalytic site. For instance, we found that the residues with the top 5% attention weights are concentrated in the V236–A248 region. Mutation of the residues in this region would stabilize the thermostability of this flexible loop, and along with the mutation of other high-weight residues around the catalytic site, would enhance the overall enzymatic activity (Figure 5c). Understanding the influence of distantly located residues on enzyme functions is vital for advancements in enzyme engineering. By targeting the modifications of those regions outside the catalytic site, enzyme activity, stability, and specificity may be modulated without directly altering the core catalytic site, resulting in enzyme inactivation. However, experimentally or simulation investigating these effects poses significant challenges that often require extensive mutagenesis studies and cost. Thus, our BioStructNet model would help to identify the distal residues as candidates for further evaluation, significantly reducing the cost of screening a large mutant database.

**Figure 6:**
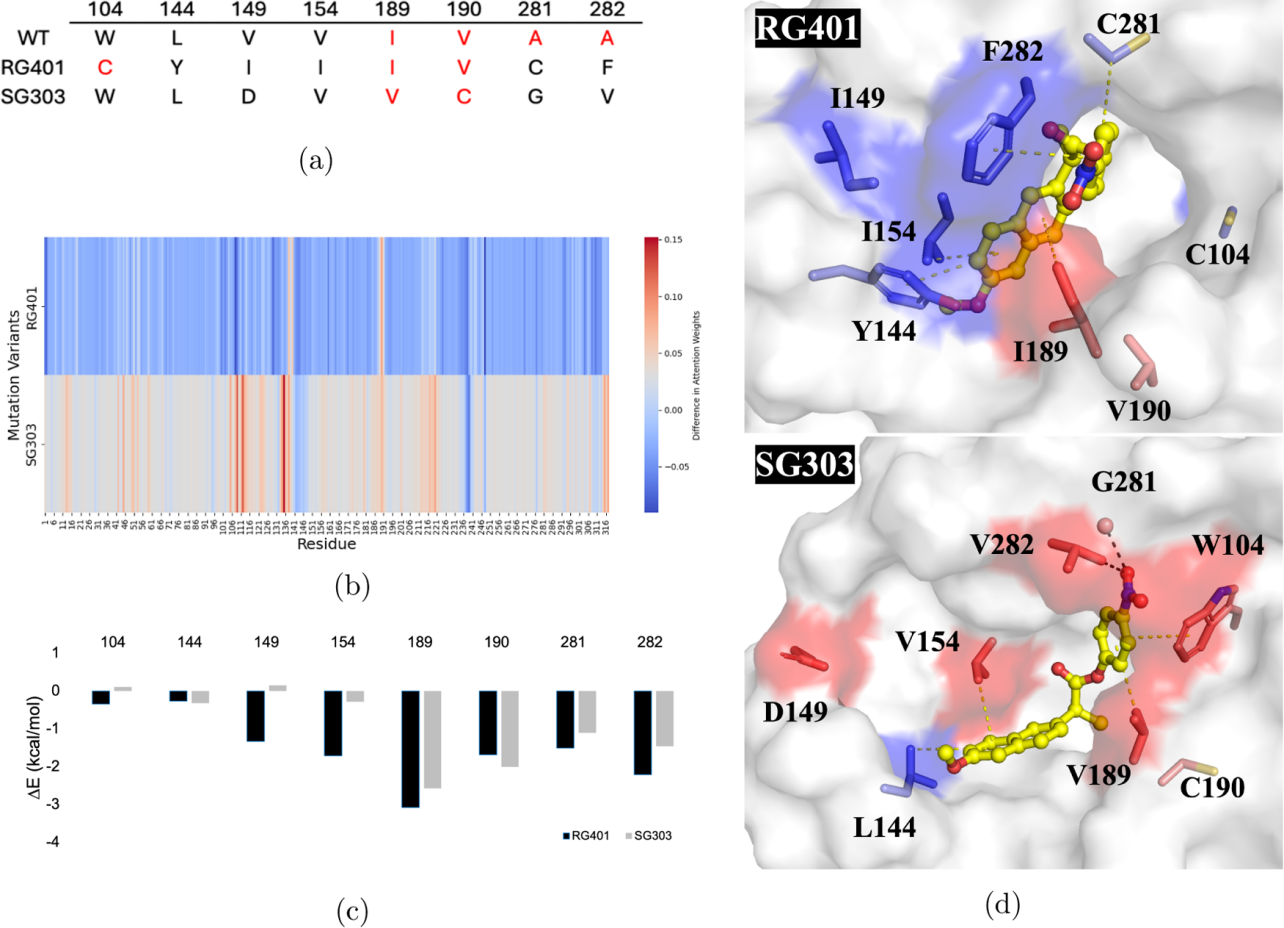
Comparison between two CalB variants RG401 and SG303 with the wild-type CalB enzyme. (a) Mutation sites in the variants. The residues with high attention weight changes compared to the wild-type are shown in red. (b) A comparison heat map of variants RG401 and SG303 that reflects the differences in attention weights at each site (or residue) between the variants and wild-type caused by mutations. The color in the heat map indicates the magnitude of the difference in attention weights. Colors closer to red (positive difference) or blue (negative difference) signify greater differences in attention weights. (c) Binding energy decomposition of critical residues in RG401 and SG303 during MD simulations, performed using the molecular mechanics Poisson–Boltzmann surface area (MM/PBSA) module in AMBER. (d) The three-dimensional structures of the CalB variants RG401 and SG303 in complex with the substrate rac-10. The substrate is represented by a yellow ball-and-stick model. The mutation residues are colored based on their respective attention weights predicted by machine learning. Residues with attention weight changes compared with the wild-type are shown in red (indicating a positive difference) or blue (indicating a negative difference). Hydrophobic interactions are represented by yellow dashed lines, while van der Waals contacts are represented by black dashed lines.

We also examined two CalB variants, RG401 and SG303, which were reported to show distinct enantioselectivity.^38^ Compared with the wild-type protein, RG401 has the mutations W104C, L144Y, V149I, V154I, A281C, and A282F; while SG303 has the mutations V149D, I189V, V190C, A281G, and A282V (Figure 6a). The attention weights of the RG401 and SG303 variants were compared with that of the wild-type, and the heat maps were plotted, which reflects the changes of attention weights at each residue position introduced by combined mutations (Figure 6b). Red indicates a positive difference in attention weights in the variants compared to the wild-type, while blue represents a negative difference, with the darker color highlighting regions with notable changes in attention. The heat map of the RG401 variant displays a significant negative difference in overall attention weights, while the heat map of the SG303 variant exhibits a more complex pattern with both positive and negative differences at different residue positions. We then compared the structures of these variants in complex with those of the rac-10 ligand (Figure 6d). Residues 189 and 190 exhibit positive attention weight changes and residue 144 shows negative changes in both SG303 and RG401 variants, indicating these three positions are responsible for the enhanced yield in the variants compared to the wild-type. The opposite enantioselectivity of the RG401 and SG303 variants may be attributed to the opposite direction of attention weight shifts in the first and second sphere residues. Specifically, residues 149, 154, 281, and 282, which are located in the substrate binding pockets, exhibit opposite shifts in attention weights in the two variants (Figure 6b). These differential shifts suggest that mutations at these sites reshape their substrate binding pockets, causing the substrate to adopt opposite binding orientations in the two variants, which ultimately leads to the production of opposite enantiomers (Figure 6d). Per-frame protein–substrate interaction fingerprints analysis was conducted using ProLIF^40^ based on the MD simulated structures of mutant enzyme complexes (Supporting Figure S7). These fingerprints quantitatively depict protein–ligand interactions. Residues such as Y144/I154/I189/C281/F282 in RG401 and W104/L144/V154/V189/G281/V282 in SG303, exhibit pronounced hydrophobic interactions, these findings align with the residue contribution shown in the heat map predicted by BioStructNet (Figure 6b).

We calculated the binding energies by the MM/PBSA (Figure 6c) based on MD simulation of the complex structures and compared them with the attention weights from BioStructNet. The catalytic residues S105 and A281 and A282 are identified, in alignment with the observed residue contribution in protein–ligand interactions identified from the heat map residue contribution. Specifically, residues like I149, I154, I189, V190, C281 and F282 in the RG401 variant and V189, C190, G281, and V282 in the SG303 variant exhibit significant energy contributions to ligand binding. This demonstrates the model’s capability for predicting protein–ligand interactions. The comparative analysis between wild-type and mutated enzymes (RG401 and SG303) further revealed significant differences in attention weights across various residues and the impact of mutations resulting in different protein–ligand bindings, thereby validating the model performance in predicting the protein–ligand interactions responsible for their functional changes.

## Conclusions

In this study, we developed BioStructNet, a novel deep learning framework tailored for predicting compound-protein interactions (CPIs), with a particular focus on small biocatalyst data sets with specific functions, such as CalB. By leveraging protein’s structural data and advanced graph-based representations, BioStructNet effectively captures both local and global interaction patterns between enzymes and substrates, offering a significant improvement over traditional methods in predicting enzymatic functions.

BioStructNet uses two different interaction modules, namely, the Bilinear Co-attention Network (BCN) and a Transformer-based module, to enhance the model’s interpretability and predictive power. The BCN module, with its bilinear attention mechanism, demonstrated its strength in detecting subtle local interactions, particularly useful in processing data sets with high structural similarity. The Transformer-based module, by contrast, provided insight into long-range and global interactions within protein–ligand complexes through its self-attention and cross-attention mechanisms. The inclusion of layer normalization and a feed-forward network further improved training stability and model performance (Supporting Figure S11).

BioStructNet, when combined with transfer learning, significantly outperforms traditional models in predicting enzyme catalytic activities with small data sets. By pretraining the model on large data sets of enzyme–substrate interactions and fine-tuning on small, function-specific data sets, overfitting—a common challenge in small data sets was tackled, while the model’s robustness and accuracy were improved. BioStructNet also enabled the identification of critical residues, including those distant from the active site, which may play important roles in enzyme function through allosteric effects or contributions to stabilizing enzyme structures. The CPI prediction in BioStructNet was validated by molecular docking combined with molecular dynamics simulations. The BioStrucNet provides a comprehensive framework for predicting and understanding protein–ligand interactions. Particularly, the attention mechanism facilitates the validation of the CPI predictions by simulated ligand protein complexes, making it a powerful tool for guiding enzyme engineering and biocatalyst discovery.

BioStructNet provides a robust and interpretable method for predicting enzyme–substrate interactions, effectively integrating structural representations, and utilizing transfer learning techniques. It would serve as a valuable tool for guiding enzyme engineering efforts with potential applications in environmental biotechnology and beyond. The successful application of BioStructNet for predicting the functions of small target data set would lay the ground framework for further model refinement and these data sets exploitation across diverse biochemical applications.

## Methods

### Data Collection and Preprocessing

We collected comprehensive databases of enzyme–substrate interactions encompassing a wide range of enzyme functions and substrates. The data included protein structures, compound structures, and the associated interaction properties.

#### Collection and Preparation of the *K*_cat_ Data Set

The source task *K*_cat_ data set for constructing the deep learning model was selectively extracted from the BRENDA^41^ and SABIO-RK^42^ databases using customized scripts through their respective application programming interfaces (APIs). Only enzymes with EC numbers beginning with “3” were included, focusing on those involved in hydrolase activities. As the majority of *K*_cat_ values reported in BRENDA and SABIO-RK do not specify their assay conditions, such as pH and temperature, these features were not included to maintain the training data set size and variety. In addition, substrate SMILES, a string notation representing the substrate structure, was extracted by querying the PubChem compound database^43,44^ with substrate names, followed by a Python-based script to ensure the canonical SMILES representation of substrates. Several rounds of data cleaning were performed to ensure the quality. Protein sequences were queried with two methods: for entries with Uniprot ID information,^45^ the amino acid sequences could be obtained via the application programming interface of the Uniprot with the help of Biopython v.1.78 (https://biopython.org/); and for entries without Uniprot ID, amino acid sequences were acquired from the Uniprot and the BRENDA databases based on their EC number and organism information. The Uniprot ID was used to search the substrate information for each sequence. Subsequently, the UniProt ID was utilized to retrieve the corresponding PDB IDs for each sequence from the ExPASy database,^46^ and only the data with structure information are retained. From the Protein Data Bank,^47^ corresponding PDB IDs are downloaded, and if the structure is complex, the ligand is deleted, retaining only chain A. Subsequent mutations are modeled using Rosetta^48^ to predict structures, ensuring that if the sequence from the structure file does not match the sequence from UniProt, the sequence from the structure file is used. The final source task data set, formed for a regression task, consists of *K*_cat_ data (in log2 scale) for enzymes with EC numbers beginning with “3” comprising 1664 items with 244 unique PDB structures. The data processing procedure was modified from previous methods,^19,49^ and a detailed explanation of the process diagram, including specific data cleaning steps, can be found in Supporting Figure S8.

#### Collection and Preparation of the Human Data Set

Created by Liu et al.,^50^ this data set includes highly credible negative samples of compound-protein pairs obtained by using a systematic screening framework containing 6728 items. We used a balanced data set cleaned by previous studies, where the ratio of positive and negative samples was 1:1.^21,35^ BioStructNet requires protein structure information; therefore, we correlate protein sequences with PDB IDs to filter and select data that include protein structural information. Finally, the human data set contains 5568 interactions and 1726 unique proteins. To compare with benchmark methods on the human data set, we employed the same partitioning method, utilizing an 80/10/10% training/validation/testing random split.

#### Collection, Molecular Dynamics Simulation, and Docking Studies of CalB and Its Variants

The conversion data for substrates by *C. antarctica* lipase B (CalB) and its mutations were collected from previous experimental reports.^37,38,51−54^ The CalB data set supports targeted research focused on optimizing and enhancing this specific activity, which falls under EC 3.1.1.3, belonging to the EC class 3. The data set contains 233 items with wild-type protein and 65 variants from published literature. SMILE strings of substrates are extracted from ZINC15.^55^ The classification models were trained and evaluated on the CalB data set, and three different threshold values (conversion ≥15, 30, 40%) were applied to partition the CalB data set for three classification tasks. Detailed information about the CalB data sets can be found in Supporting Figure S9. The cutoff values were selected based on the consideration that the 15% cutoff helps to distinguish very low conversion rates from moderate and high conversion rates, aiding in identifying cases where enzyme performance is poor or nearly nonfunctional in substrate conversion; the 30% cutoff establishes an intermediate threshold between moderate and higher conversion rates, providing a midpoint to indicate cases with moderate enzyme activity; the 40% cutoff sets a higher standard to distinguish between moderate and very high conversion rates, emphasizing cases where the enzyme performs exceptionally well. To reflect the mutation effect of CPI compared with wild-type protein, another classification rule is applied by comparing the conversion values of mutants with those of the wild-type protein for the same substrate. If a mutant’s conversion value is comparable to (taking into account experimental errors) or higher than that of the wild-type protein for the same substrate, it is classified as 1; otherwise, it is classified as 0. To enhance the robustness of model training on small data sets and reduce the impact of validation set selection on model performance, we mitigate potential negative impacts from redundant information by focusing on known mechanisms. This is achieved by setting a central point and radius to encircle the pocket area with a spherical region, thereby augmenting the data set with negative data from outside this pocket. Specifically, the catalytic reaction of the CalB enzyme occurs in an active site pocket controlled by Asp187, Ser105, and His224. These three residues form a catalytic triad, which is central to the catalytic process. We recognized that mutations in the first-sphere and second-sphere regions around these key residues could impact the enzyme’s catalytic function. However, mutations in residues far from these critical regions (i.e., non1st sphere and non2nd sphere mutations) typically have minimal impact on catalytic activity, especially when the distance exceeds 20 Å, as electrostatic interactions and other intermolecular forces are unlikely to effectively influence the active site at such distances. Based on this understanding, we devised a rational data augmentation strategy. The specific steps are as follows: First, using existing substrate information from the database and focusing on 151 residues located beyond the 20 Å range, we randomly generated a series of single-point mutations. These mutations were designed to avoid the catalytic core and secondary interaction regions while ensuring a minimal impact on catalytic activity. We generated 1208 such mutation data points and appropriately labeled them as negative samples to reflect their potentially low impact on catalytic function.

The predicted structures of CalB variants were generated using the Rosetta Comparative Modeling (RosettaCM),^48^ leveraging the crystal structure of the wild-type protein (PDB ID: 1TCA)^47^ as a template. For each variant, six candidates generated by RosettaCM were evaluated based on their free energy, and the structures with the lowest free energy were selected to build the molecular dynamics (MD) simulation systems. Each enzyme was solvated in a pre-equilibrated cuboid box of TIP3P^56^ water molecules, ensuring that any protein atom was at least 10 Å from the edge of the box. The system was neutralized by adding Na^+^ counterions by tleap module in AMBER 20.^57^ A harmonic restraint force constant of 100 kcal/mol was applied to the solute molecules and ions to minimize the solvent molecules, followed by 1000 steps of steepest descent and 1000 steps of conjugate gradient unrestrained minimization. A cutoff of 10 Å was used for nonbonded Lennard-Jones potential and electrostatic interactions. Hydrogen bonds were constrained by using the SHAKE algorithm during all MD simulations. Progressive heating was performed from 0 to 300 K over 100 ps (5000 steps with a step size of 0.02 ps) using the NVT ensemble, followed by 1 ns equilibration using the NPT ensemble at 300 K. A harmonic restraint of 5 kcal/mol was applied to the solute during the equilibration. After equilibration, a 100 ns production MD simulation was conducted using the NPT ensemble at 300 K and 1 bar. We applied cluster analysis to determine the representative protein structures of CalB variants from MD trajectories using the CPPTRAJ module^58^ in AMBER 20. Next, to prepare the functional 3D structure for docking, the represented protein structures were utilized to define the docking box and the substrate structures were prepared from SMILES using RDKit.^59^ The parameters for the model substrate were calculated based on the optimized geometry at B3LYP/6-31G(d) using Gaussian 16. The substrates were docked into the simulated proteins using AutoDock^60^ based on the previously published paper.^61,62^ The pose corresponding to the highest binding affinity score for each system was selected for subsequent MD simulations. A 100 ns MD simulation was conducted on the enzyme–substrate complex by using AMBER 20, which included steps for energy minimization and equilibration. Stable fluctuations in the root-mean-square deviation (RMSD) indicated that a stable complex structure was achieved. Principal Component Analysis (PCA)^30^ was employed to select the most representative cluster from which the stable structure was extracted.

### Model Architecture

In this section, we introduce the framework of a structure-based neural network (BioStructNet) for predicting CPI of CalB variants by transfer-learned pattern knowledge from a large related database, utilizing various data representations and methodologies to address the complexities of biochemical data (Figure 1).

#### Graph Convolution Network (GCN) for the Protein Structure

The protein structures were represented by contact map graphs, incorporating positional embeddings to maintain spatial relationships. In these protein structure graphs, nodes represent individual amino acids, each characterized by a combination of a one-hot encoding and five key physicochemical properties: molecular weight, p*K_a_*, p*K_b_*, p*K_x_*, and pI. These features offer detailed insights into each amino acid’s mass, acid and base dissociation constants, and isoelectric point, all of which are crucial for understanding their roles in protein structures and interactions. Edges between the nodes signify visual connections based on a distance map and a predefined cutoff 8 Å,^63,64^ illustrating the spatial proximity of the amino acids within the protein. This module is designed to handle protein data by leveraging the spatial information inherent in protein structures through a combination of positional encoding and graph convolution networks (GCNs). This approach allows the model to capture both local and global contextual information that is essential for understanding complex biological functions. The model begins by generating positional encodings for protein nodes, which helps in retaining sequence order in the otherwise order-agnostic GCN architecture. The positional encoding is mathematically represented by
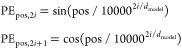
1where pos is the position, *i* runs from 0 to (*d*_model_/2) – 1, where *d*_model_ represents the dimensionality of the input feature space. This formulation allows each dimension of the positional encoding to oscillate between different frequencies, ranging from high to low, thereby uniquely encoding each position along the sequence of the protein. Following the addition of positional encodings to the node features, the model applies a series of graph convolutional layers. The GCN layers propagate information across the nodes in the graph. For each layer *l*:
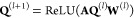
2where **A** is the adjacency matrix, **Q**^(*l*)^ is the node feature matrix at layer *l*, and **W**^(*l*)^ is the learnable weight matrix. The model sequentially applies three GCN layers, each progressively increasing the feature dimensions to capture more complex patterns in the data. After each convolution, activation functions are applied to introduce nonlinear transformations into the model, essential for learning complex functions. The output from the final GCN layer is processed through two fully connected layers to reduce the dimensionality to the desired output size. These layers consolidate features learned from the entire protein graph into a global representation.

#### GCN-GRU for the Molecular Graph

We represented the compound 2D structures as graphs with nodes denoting the individual atoms and edges denoting the chemical bonds connecting these atoms. Each atom node was represented by its chemical properties, as implemented in the DGL-LifeSci package. Each atom is represented as a 74-dimensional integer vector describing eight pieces of information: the atom type, the atom degree, the number of implicit Hs, formal charge, the number of radical electrons, the atom hybridization, the number of total Hs and whether the atom is aromatic. The combined Graph Convolutional Networks (GCNs) with Gated Recurrent Units (GRUs) module initializes a transformation for the input features to capture both spatial and sequential relationships in the ligand. The model begins with an initialization phase, where molecular features are transformed into an embedding space. We employed three GNN layers, each was customized combining with a GRU cell to aggregate neighborhood information for each node:
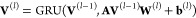
3where **A** is the adjacency matrix of the graph, **W**^(*l*)^ is the weight matrix of the *l*th layer, **b**^(*l*)^ is the bias vector of the *l*th layer, GRU is the GRU unit applied to the transformed features, and the previously hidden features. *V*^(*l*)^ represents the node feature matrix at layer *l*, where each row corresponds to the feature vector of a specific node after processing by the GRU in the *l*th layer. The message passing function aggregates messages from neighboring nodes *i* to node *j*:
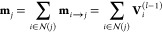
4After message passing and aggregation, a linear transformation and RELU activation function are applied. Another GRU update step combines the transformed features with the previous hidden state. Finally, the node features are reshaped to match the batch size and output dimensions.

#### BCN Interaction Module

This bilinear attention network was first designed by Bai^16^ to integrate, and process features from two distinct inputs, visual data of ligand and textual data of protein. The BCN module was modified to accept both visual data of ligand and protein with parameters that are configurable to tailor the network’s complexity and regularization. The core of these modules is the bilinear interaction, which can be mathematically represented as
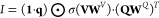
5where *V* = *v*^1^, *v*^2^, ···, *v*^*N*^ and *Q* = *q*^1^, *q*^2^, ···,*q*^*M*^ are the transformed visual features from ligand and protein, respectively, where *N* and *M* denote the number of encoded atoms in a ligand and residues in a protein. **W**^*V*^ and **W**^*Q*^ are weight matrices for the *i*th bilinear interaction. **q** is a learnable weight vector, and **1** is a fixed all-ones vector. *I* indicates the interaction intensity of respective pairs. ⊙ denotes the Hadamard (element-wise) product and σ denotes the activation function. An element *I*_*i*,*j*_ in eq 5 can be written as
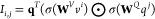
6where: *v*^*i*^ is the *i*th substructural representation of the ligand, and *q*^*j*^ is the *j*th substructural representation of the protein. This bilinear form projects the features into a joint embedding space, facilitating a comprehensive interaction analysis. Following the computation of interaction tensors, the module applies pooling to summarize the spatial information effectively and obtain the *k*th element of joint representation *f*_*k*_^′^ as
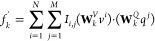
7where **W**_*k*_^*V*^ and **W**_*k*_^*Q*^ denote the *k*th column of weight matrices. Finally, sum pooling is added to the joint representation vector to obtain a compact feature map:
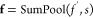
8where the SumPool function is a one-dimensional and nonoverlapped sum pooling operation with stride *s*.

#### Transformer-Based Interaction Module

The method encapsulates a neural network architecture that leverages attention mechanisms to process and integrate protein and ligand features. The TransformerBlock first applies self-attention to the protein features to capture dependencies within the protein sequence. This mechanism can be represented as
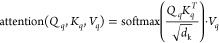
9where *Q*_*q*_, *K*_*q*_, and *V*_*q*_ represent the queries, keys, and values derived from the protein and ligand features through the multihead attention mechanism. Specifically, for protein features, queries, keys, and values are obtained by linearly projecting the input feature vectors into different subspaces. In multihead attention, these projections are used to compute attention scores (from queries and keys) and retrieve context-aware features (from values), so as to capture both intraprotein and cross-protein–ligand interactions. *d*_k_ is the dimensionality of the keys. This operation enhances the representation of protein features by emphasizing informative parts of the sequence. A normalization layer follows this step to stabilize learning by normalizing the layer outputs. Following the self-attention layer, a cross-attention mechanism is employed:
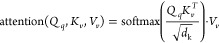
10Here, the updated protein features *Q*_*q*_ serve as queries, while both keys *K*_*v*_ and values *V*_*v*_ are derived from the transformed ligand features. This cross-attention step allows the model to dynamically focus on how parts of the protein sequence relate to the ligand features, facilitating a deeper understanding of their interaction. The output of this layer also undergoes normalization.

The outputs from the cross-attention layer are then passed through a feed-forward network (FFN), which typically consists of two linear transformations with a nonlinear activation function in between:
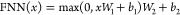
11This layer further processes the features to refine the representation for the prediction tasks. Finally, a global average pooling operation reduces the feature dimension by averaging across the sequence dimension, which simplifies the output while retaining the essential information. The pooled features are then fed into a final linear layer to produce the model’s output, suitable for downstream tasks such as classification or regression.

#### Training and Evaluation

The training process involves pretraining the general model on a large-scale data set of enzyme–substrate interactions and fine-tuning it on smaller, function-specific data sets to improve prediction accuracy for specific enzyme functions. This transfer learning approach leverages the broad knowledge captured by the general model and adapts it to specific tasks of interest. For the source task data set, a 5-fold validation method is employed. This approach is chosen because it allows for a robust estimate of the model’s performance by averaging the results from five different partitions. This helps ensure that the model is tested thoroughly across different subsets of data, providing a reliable measure of its predictive power and generalization ability. For the smaller CalB data set, bootstrapping is used as the method of validation. Bootstrapping is particularly useful for assessing the stability and generalization ability of models, especially when the sample size is limited. Early stopping is implemented, where training automatically halts if there is no improvement in the performance on the validation set for five consecutive epochs. This strategy helps prevent overfitting and conserves computational resources, making it highly effective for managing smaller data sets. To evaluate the performance of the developed models, a comprehensive set of metrics was employed, including root-mean-square error (RMSE), coefficient of determination (*R*^2^), accuracy, AUC, and relative error (RE). RMSE is a widely used metric for evaluating the accuracy of regression models. It is defined as the square root of the average of the squared differences between the predicted and actual values:
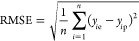
12where *y*_*i*p_ is the predicted value, *y*_*i*e_ is the experimental value. If the predicted responses are sufficiently close to the true values, then the RMSE would be small. On the contrary, if the predicted and true responses differ substantially, the RMSE would be large. *R*^2^ provides a measure of how well future samples are likely to be predicted by the model:
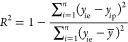
13where *y̅* is the average of the experimental values and *n* is the total number of items in the data set (validation data set or test data set). *R*^2^ indicates the proportion of the variance in the dependent variable that is predictable from that of the independent variables.

Accuracy is the proportion of true results (both true positives and true negatives) among the total number of cases examined, which is a widely used metric to evaluate the performance of binary classification models:
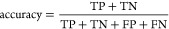
14where TP, TN, FP, and FN represent the number of true positives, true negatives, false positives, and false negatives, respectively. In the context of classification, RE is used to assess the model’s accuracy in estimating the probability of the positive class. RE for a given threshold is defined as
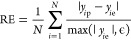
15where *y*_*i*p_ is the predicted probability of the positive class, *y*_*i*e_ is the actual class label (0 or 1), *N* is the number of samples, and ϵ is a small constant to avoid division by zero. This metric is relevant at 1.0% probability thresholds (e.g., 0.01, 0.02, ···, 1.00) to assess the sensitivity of the model’s predictions upon trivial changes in the decision boundary. It helps in identifying how well the model’s probability estimates align with true class labels under different operational settings.

#### Fine-Tuning Models

In this study, we explored the effectiveness of three distinct fine-tuning methodologies applied to two different interaction modules, BCN and transformer-based interaction modules. This resulted in a total of six unique model configurations, designed to investigate how different fine-tuning strategies influence the performance of interaction modules in capturing complex biochemical interactions. The fine-tuning methods employed are described as follows:

##### Free-Layer Fine-Tuning

Unlike the blocked method, free-layer fine-tuning allows for the adjustment of all layers within the interaction modules. This method offers the flexibility to adaptively learn from new data across the entire depth of the model architecture, potentially capturing more nuanced patterns and dependencies than more restrictive approaches.

##### Blocked Layer Fine-Tuning

This method involves fine-tuning the output layers within the interaction modules while keeping the remaining layers frozen. By isolating the adjustments to certain layers, this approach aims to refine model responses without extensive retraining of the entire network. It was applied independently to both the BCN and the transformer-based modules.

##### LoRa Fine-Tuning

The low-rank (LoRa) fine-tuning method^65^ focuses on optimizing the last layer of the interaction modules, using low-rank matrix adjustments to modify the internal representations within a neural network layer, thereby enhancing model performance while maintaining consistent input and output dimensions. In the transformer-based interaction module, the LoRa fine-tuning method is applied after the attention mechanism. The attention logits are computed solely based on the attention mechanism, and then the adjustments are made to the final layer to refine the model’s output. Similarly, in the BCN interaction module, LoRa fine-tuning is applied after the attention mechanism. However, since the logits in BCN are generated using both the attention outputs and the original ligand and protein inputs, LoRa adjustments are applied separately to the ligand and protein inputs. This dual adjustment ensures that the fine-tuning process captures the intricate interactions between the ligand and protein more effectively.

### Experimental Setting

#### Implementation

BioStructNet is implemented in Python 3.10, along with functions from torch 2.2.2,^66^ Biopython 1.81,^67^ DGL-LifeSci 0.3.2,^68^ Scikit-learn 1.3.0,^69^ Numpy 1.26.4^70^ and Pandas 2.0.3.^71^ For the initial models, the batch size is set to be 64 and the Adam optimizer is used with a learning rate of 5 × 10^–5^. We configured the model to use 5-fold cross-validation with each fold running for a maximum of 100 epochs. For the transfer learning models, we employed a bootstrapping approach with 100 iterations to ensure a robust performance estimation. Early stopping was implemented with a patience of five epochs to prevent overfitting and conserve computational resources. For each bootstrap iteration, the model’s performance was monitored at every epoch on the validation set, tracking metrics such as accuracy, AUC, precision, recall, and other relevant indicators. The best epoch within each bootstrap iteration, determined by the highest AUC score, was selected. The final performance of the models was then calculated by averaging the metrics across all bootstrap iterations. This approach ensures that the evaluation captures the variability of the model’s predictions, providing a reliable estimate of its performance for classification tasks. The protein feature encoder consists of three GCN layers with a number of filters [128, 128, and 128]. The ligand feature encoder consists of three GCN layers with hidden dimensions [128, 128, and 128] as well. In the bilinear attention module, we employ only two attention heads to provide better interpretability. The latent embedding size is set to 768 and the sum pooling window size is 3. The number of hidden neurons in the fully connected decoder is 512. In the transformer-based interaction module, it is initialized with a model dimension of 128 and utilizes 2 attention heads. The dimension through a feed-forward network (FFN) increases from 128 to 512 and then reduces back to 128. Our model performance is not sensitive to hyperparameter settings. The configuration details and sensitivity analysis are provided in Supporting Table S3 and Figure S10. To thoroughly analyze the contributions of key components in BioStructNet, we designed and conducted ablation experiments focusing on attention mechanisms and structural information integration. The ablation experiment results show larger RMSE values and instances where the *R*^2^ score drops below 0, highlighting the importance of the key components (Supporting Figure S11).

#### Baselines

We compare BioStructNet with the other models on compound-protein interaction prediction: The shallow machine learning methods—random forest (RF), *k*-nearest neighbors (KNN) and (L2)—are applied to analyze protein and ligand graph node features aggregated using global sum pooling; Tsubaki’s^35^ and DLKcat^19^ used GNNs to update the atom vectors of molecular graphs, and CNNs to scan the *n*-gram split protein sequences, respectively suited for classification and regression tasks; DrugVQA^21^ is a protein structure-based method employing a dynamic attentive CNN to handle variable-length distance maps of proteins and a self-attentional sequential model to extract semantic features from molecules; TransformerCPI2.0^36^ utilizes TAPE-BERT for protein sequence representation with a self-attention-based transformer encoder and introduces a virtual atom vector to enhance molecular interaction information; ALDELE^20^ is based on five distinct toolkits to integrate sequence-based features for proteins and structure-based physicochemical features of ligands, along with a two-phase attention neural network to predict the interactions; DrugBAN^16^ employs a dual-encoder architecture combining CNNs for protein sequences and GCNs for molecules, which are then combined using a bilinear attention network to capture pairwise local interactions. DrugVQA and DrugBAN, originally developed for classification tasks, were adapted for regression in this study by modifying their output layer. For these baseline models, we adopted the recommended hyperparameter configurations, as outlined in their respective original publications, to ensure reproducibility and consistency.

## Data Availability

Source code, original data, and instructions are available at: https://github.com/Xiangwen-Wang/BioStructNet.

## Supporting Information Available

Results of BioStructNet fine-tuning models based on a transformer-based model on the CALB data set (Table S1), experimental results of both conversion and *K*_cat_ values for wild-type CalB protein (PDB ID: 1TCA) and variants with different substrates (Table S2), hyperparameter configuration in BioStructNet (Table S3), BioStructNet performance on the BindingDB *K*_d_ data set (Figure S1), molecular structure of “rac-5”, “rac-7”, “rac-8”, “rac-10”, and “rac-12” (Figure S2), RMSD analysis of the MD simulated structures of CalB and its variants in complex with different substrates (Figure S3), conformational distributions of the protein–ligand complex ensembles from PCA analysis of MD simulations (Figure S4), machine learning attention heat maps of the CalB-substrate complexes (Figure S5), 2D interaction maps around the substrate binding pocket in CalB (Figure S6), detailed per-frame protein–substrate interaction fingerprints generated using ProLIF for mutant enzymes interacting with the ligand (Figure S7), data collecting and cleaning and the distribution of the *K*_cat_ data set for enzymes involved in hydrolase activities (with EC number begin with “3”) (Figure S8), detailed information on CalB conversion data set (Figure S9), learning curves of RMSE and *R*^2^ with various hyperparameters on the *K*_cat_ validation set (Figure S10), and learning curves of RMSE and *R*^2^ of ablation experiments on the *K*_cat_ data set (Figure S11) (PDF)
