# Supplementary material for: BioStructNet: Structure-Based Network with Transfer Learning for Predicting Biocatalyst Functions
Source: J Chem Theory Comput. 2024 Dec 20;21(1):474–90. doi: 10.1021/acs.jctc.4c01391 (PMC11736791; doi:10.1021/acs.jctc.4c01391)
Supplement: Supplementary file 1 — ct4c01391_si_001.pdf [file ct4c01391_si_001.pdf]

BioStructNet: Structure-Based Network with Transfer Learning for Predicting Biocatalyst Functions

Xiangwen Wang[1,2], Jiahui Zhou[1], Jane Meuller[2], Derek Quinn[2], Alexandra Carvalho[2], Thomas S. Moody[2,3], and Meilan Huang[1,*]

[1]School of Chemistry and Chemical Engineering, Queen's University Belfast, BT9 5AG, Northern Ireland, U.K.

[2]Department of Biocatalysis and Isotope Chemistry, Almac Sciences, Craigavon, BT63 5QD, Northern Ireland, U.K.

[3]Arran Chemical Company Limited, Unit 1 Monksland Industrial Estate, Athlone, Co. Roscommon, N37 DN24, Ireland

*Corresponding e-mail: m.huang@qub.ac.uk

Table of Contents

Table S1 The results of BioStructNet fine-tuning models based on transformer-based model on CALB dataset. 3

Table S2 Experimental results of both conversion and Kcat values for wild-type CalB protein (PDB ID: 1TCA) and variants with different substrates. 4

Table S3 Hyperparameter configuration in BioStructNet 5

Figure S1 The BioStructNet performance on BindingDB Kd dataset.. 6

Figure S2 The molecular structure of "rac-5", "rac-7", "rac-8", "rac-10" and "rac-12". 7

Figure S3 The RMSD analysis of the MD simulated structures of CalB and its variants in complex with different substrate. 8

Figure S4 The conformational distributions of protein−ligand complex ensembles from PCA analysis of MD simulations. 9

Figure S5 The machine learning attention heat maps of the CalB-substrate complexes. 11

Figure S6 The 2D interaction maps around the substrate binding pocket in CalB.. 14

Figure S7 Detailed per-frame protein-substrate interaction fingerprints generated using ProLIF for mutant enzymes interacting with the ligand. 15

Figure S8 Data collecting and cleaning and the distribution of the Kcat dataset for enzymes involved in hydrolase activities (with EC number begin with "3"). 16

Figure S9 The detailed information of CalB conversion dataset.. 17

Figure S10 Learning curves of RMSE and R2 with various hyperparameters on the Kcat validation set.. 18

Figure S11 Learning curves of RMSE and R2 of ablation experiments on the Kcat dataset.... 19

Table S1 The results of BioStructNet fine-tuning models based on transformer-based model on CALB dataset.

**Cutoff**	**TL Models**	***AUC***	***Accuracy***	***1.0% RE***
15	Block	81.25%	0.60	0.19
	Free	81.39%	0.59	0.19
	LoRa	80.79%	0.67	0.24
30	Block	61.86%	0.56	0.42
	Free	62.66%	0.57	0.37
	LoRa	62.10%	0.58	0.45
40	Block	59.00%	0.60	0.46
	Free	54.56%	0.59	0.44
	LoRa	60.15%	0.60	0.46

Table S2 Experimental results of both conversion and Kcat values for wild-type CalB protein (PDB ID: 1TCA) and variants with different substrates.

Protein name*	Mutants	Substrate name*	Substrate smiles	Conversion (%)	Kcat (s-1)
WT	WT	rac-10	COc1ccc2cc(C(C)C(=O)Oc3ccc([N+](=O)[O-])cc3)ccc2c1	9	0.05
WT	WT	rac-5	Cc2ccc(C(C)C(=O)Oc1ccc(N(=O)=O)cc1)cc2	39	0.18
WT	WT	rac-7	CC(C(=O)Oc1ccc([N+](=O)[O-])cc1)c1ccc(-c2ccccc2)c(F)c1	13	0.09
WT	WT	rac-8	CC(C(=O)Oc1ccc([N+](=O)[O-])cc1)c1cccc(C(=O)c2ccccc2)c1	31	0.11
WT	WT	rac-12	CCCCC(CC)C(=O)Oc1ccc([N+](=O)[O-])cc1	14	0.05
RG401	WA104C/LA144Y/VA149I/VA154I/AA281C/AA282F/	rac-10	COc1ccc2cc(C(C)C(=O)Oc3ccc([N+](=O)[O-])cc3)ccc2c1	19	0.82
RG401	WA104C/LA144Y/VA149I/VA154I/AA281C/AA282F/	rac-12	CCCCC(CC)C(=O)Oc1ccc([N+](=O)[O-])cc1	23	4.57
RG401	WA104C/LA144Y/VA149I/VA154I/AA281C/AA282F/	rac-5	Cc2ccc(C(C)C(=O)Oc1ccc(N(=O)=O)cc1)cc2	45	0.8
RG401	WA104C/LA144Y/VA149I/VA154I/AA281C/AA282F/	rac-7	CC(C(=O)Oc1ccc([N+](=O)[O-])cc1)c1ccc(-c2ccccc2)c(F)c1	12	0.6
RG401	WA104C/LA144Y/VA149I/VA154I/AA281C/AA282F/	rac-8	CC(C(=O)Oc1ccc([N+](=O)[O-])cc1)c1cccc(C(=O)c2ccccc2)c1	6	1.66
SG303	V149D/I189V/V190C/A281G/A282V	rac-10	COc1ccc2cc(C(C)C(=O)Oc3ccc([N+](=O)[O-])cc3)ccc2c1	30	0.2
SG303	V149D/I189V/V190C/A281G/A282V	rac-12	CCCCC(CC)C(=O)Oc1ccc([N+](=O)[O-])cc1	49	0.01
SG303	V149D/I189V/V190C/A281G/A282V	rac-5	Cc2ccc(C(C)C(=O)Oc1ccc(N(=O)=O)cc1)cc2	54	0.1
SG303	V149D/I189V/V190C/A281G/A282V	rac-7	CC(C(=O)Oc1ccc([N+](=O)[O-])cc1)c1ccc(-c2ccccc2)c(F)c1	21	0.24
SG303	V149D/I189V/V190C/A281G/A282V	rac-8	CC(C(=O)Oc1ccc([N+](=O)[O-])cc1)c1cccc(C(=O)c2ccccc2)c1	31	0.09

* The names of the proteins and substrates are taken from the original papers.

Table S3 Hyperparameter configuration in BioStructNet

Module	Hyperparameter	Values
Protein encoder	Protein contact map	Cα
	Initial amino acid embedding	128
	Hidden node dimensions	[128, 128, 128]
	Dropout	0.2
Ligand encoder	Initial atom embedding	128
	Hidden node dimensions	[128, 128, 128]
BCN interaction	Heads of BCN attention	2
	Sum pooling window size	3
	Dropout	0.2
Transformer-based	Heads of self-attention	2
	Heads of cross-attention	2
	Hidden dimension	128
MLP	Hidden dimension	512
Solver	Learning rate	5e-5
	Epoch	100

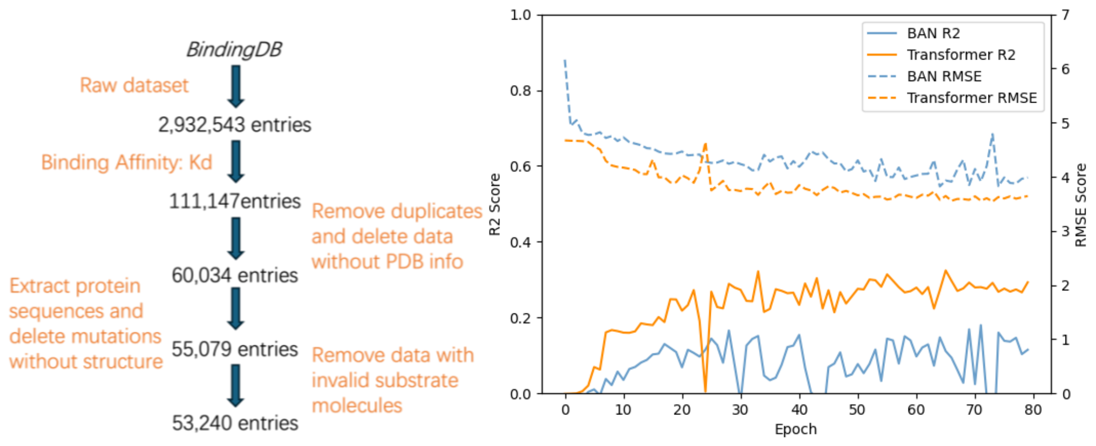

Figure S1 The BioStructNet performance on BindingDB Kd dataset. **a).** Data collecting and cleaning process for BindingDB Kd dataset. **b).** Learning curves of RMSE and R2 on BindingDB Kd dataset.

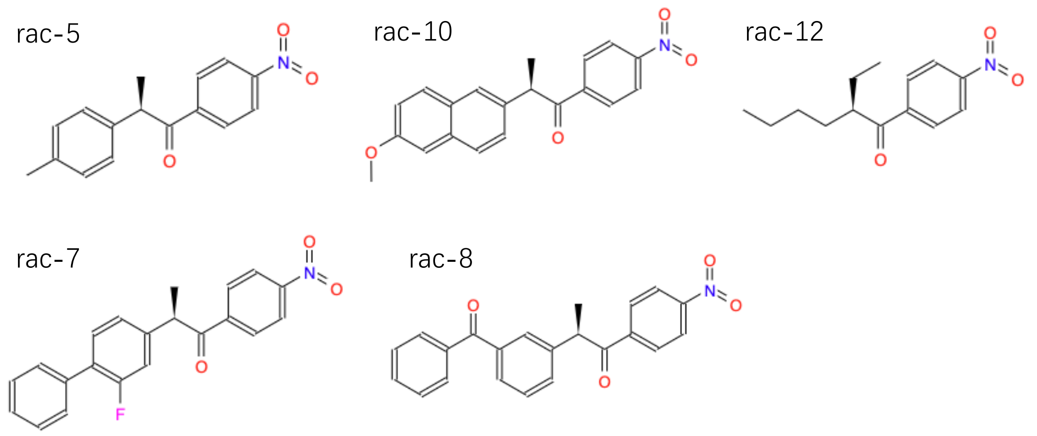

Figure S2 The molecular structure of “rac-5”, “rac-7”, “rac-8”, “rac-10” and “rac-12”.

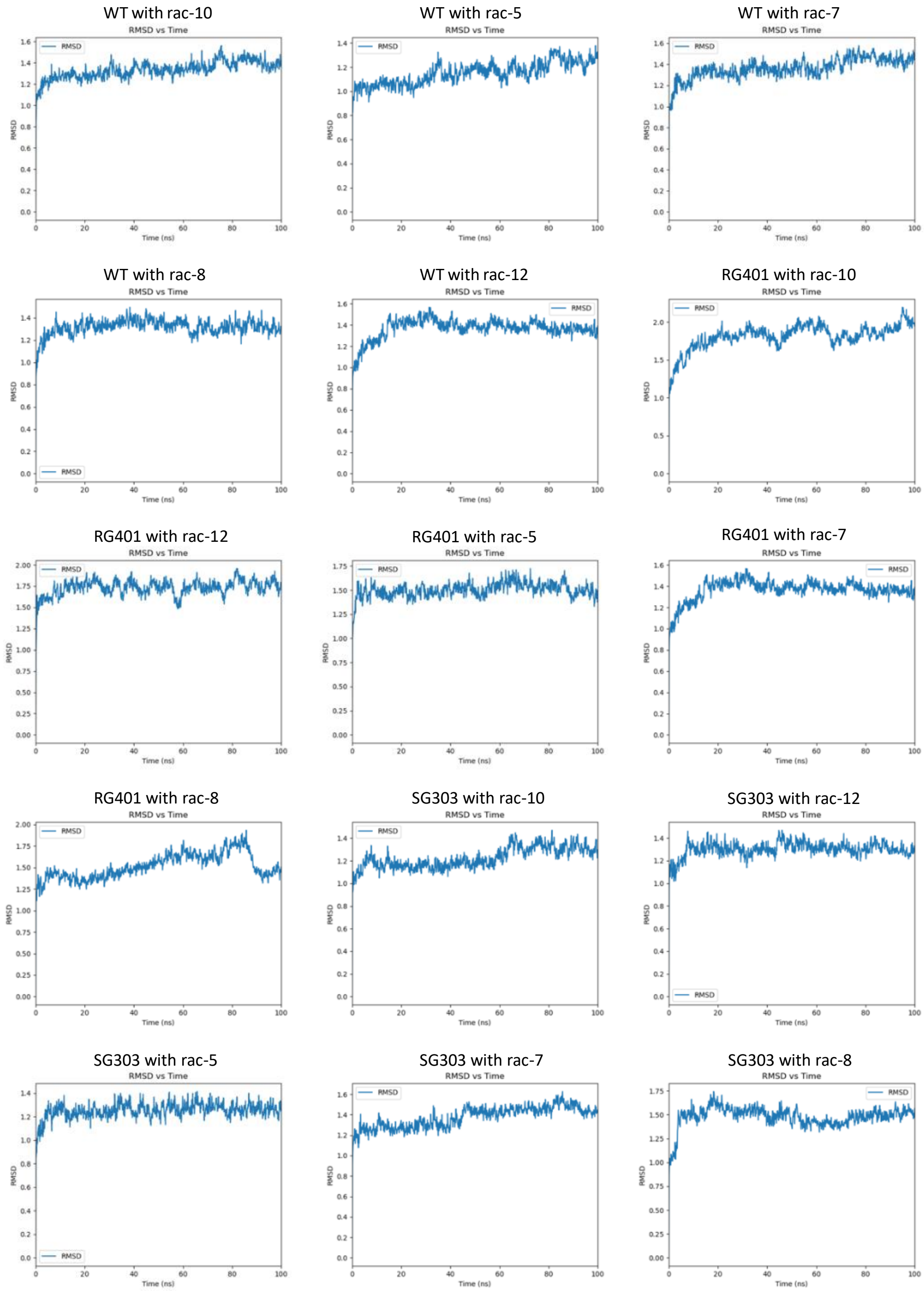

Figure S3 The RMSD analysis of the MD simulated structures of CalB and its variants in complex with different substrate.

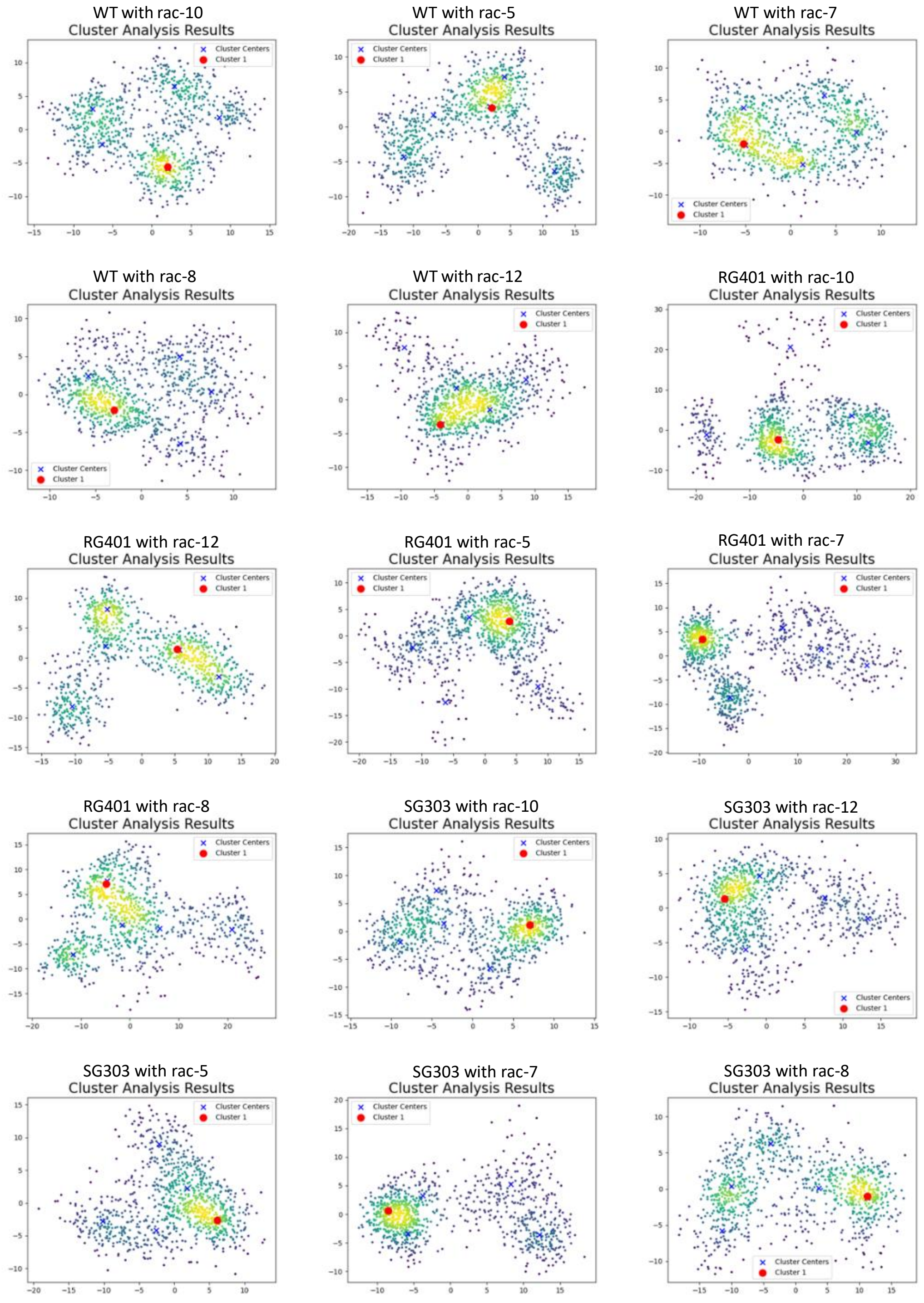

Figure S4 The conformational distributions of protein−ligand complex ensembles from PCA analysis of MD simulations.

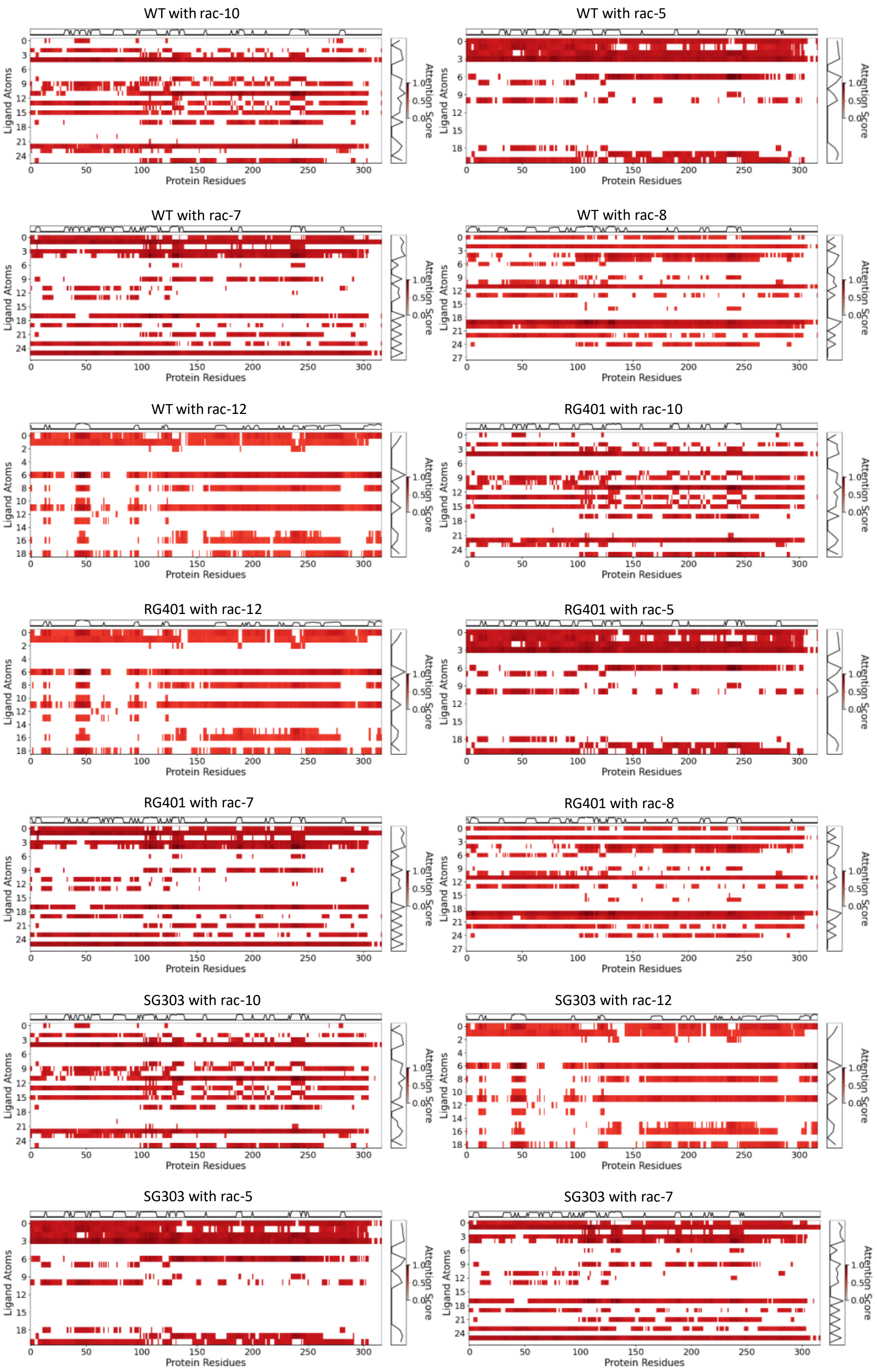
WT with rac-10
WT with rac-5
WT with rac-7
WT with rac-8
WT with rac-12
RG401 with rac-10
RG401 with rac-12
RG401 with rac-5
RG401 with rac-7
RG401 with rac-8
SG303 with rac-10
SG303 with rac-12
SG303 with rac-5
SG303 with rac-7
Ligand Atoms
Protein Residues
Attention Score

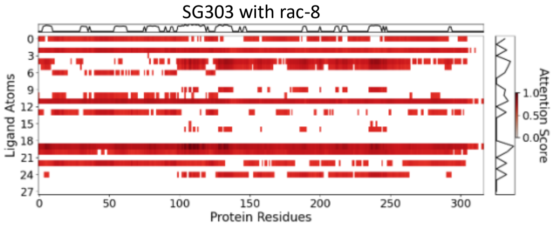

Figure S5 The machine learning attention heat maps of the CalB-substrate complexes.

WT with rac-10

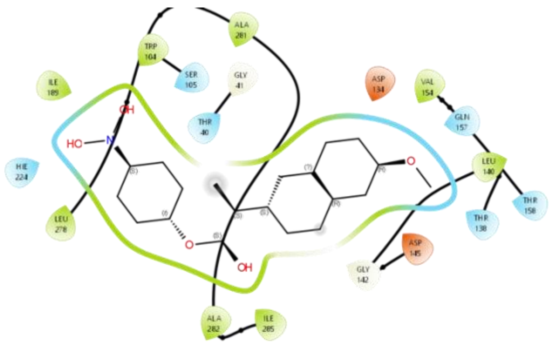

WT with rac-5

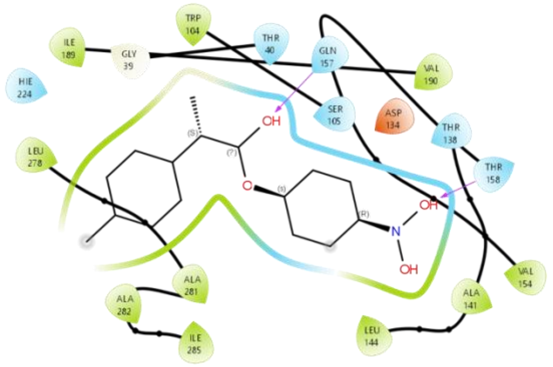

WT with rac-7

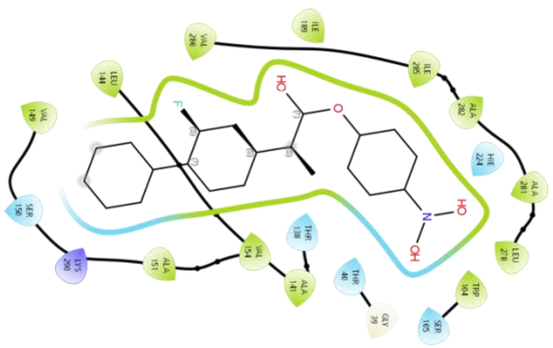

WT with rac-8

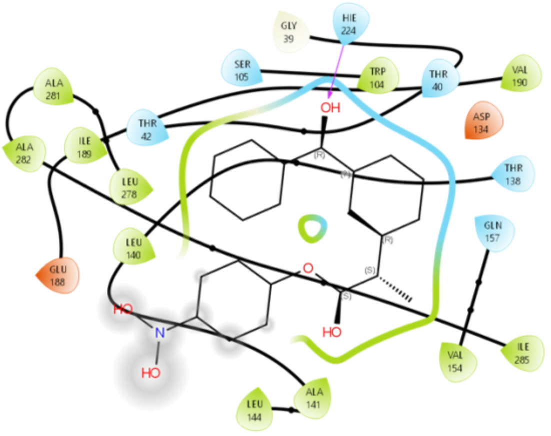

WT with rac-12

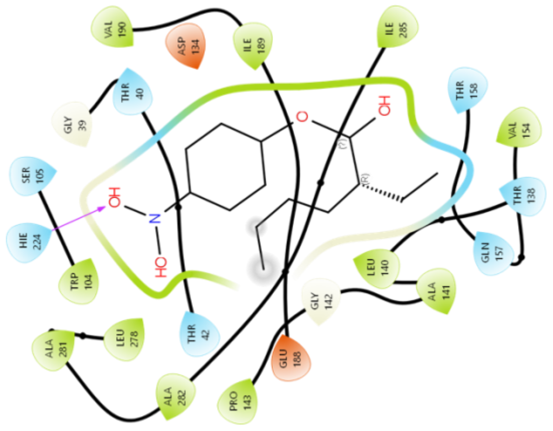

RG401 with rac-10

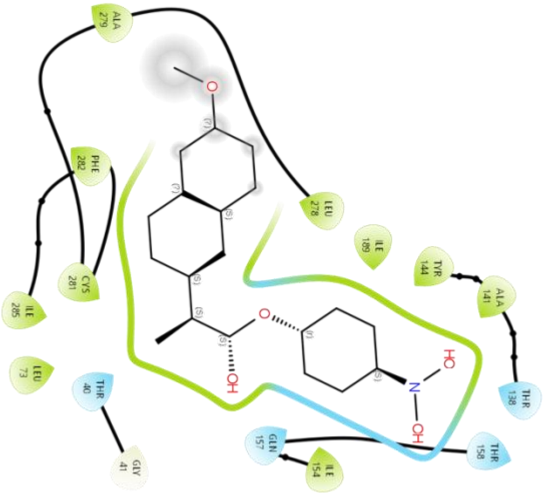

RG401 with rac-12

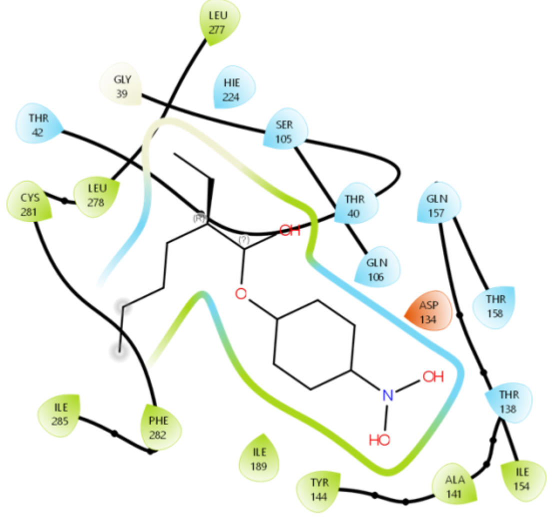

RG401 with rac-5

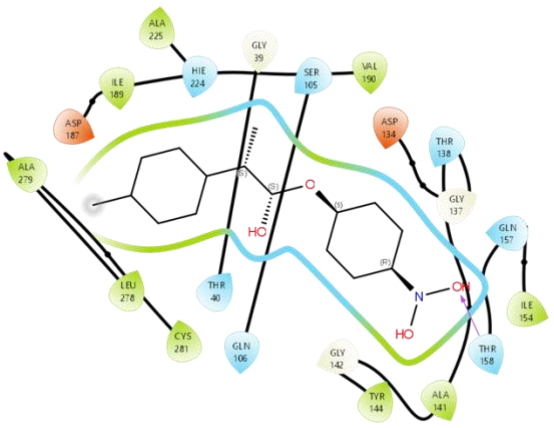

RG401 with rac-7

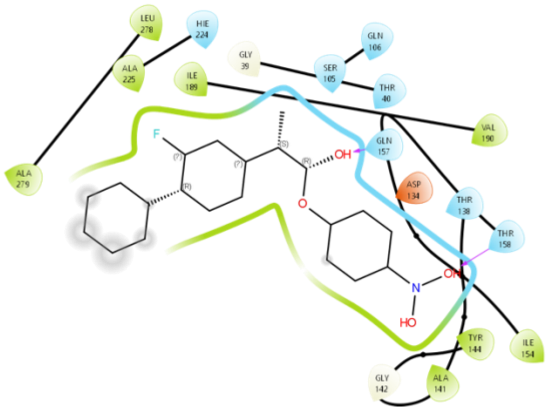

RG401 with rac-8

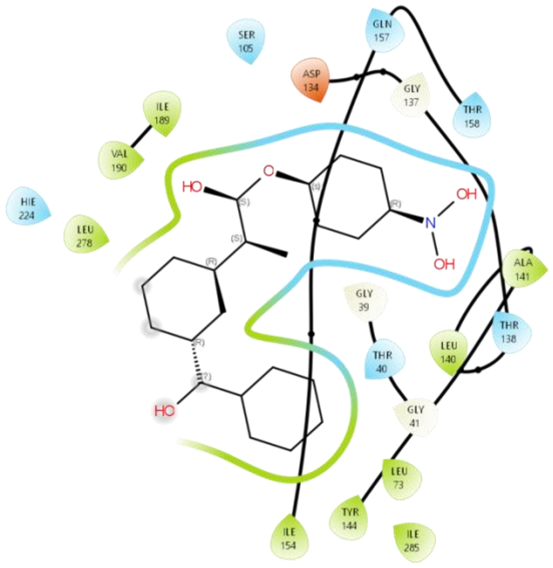

SG303 with rac-10

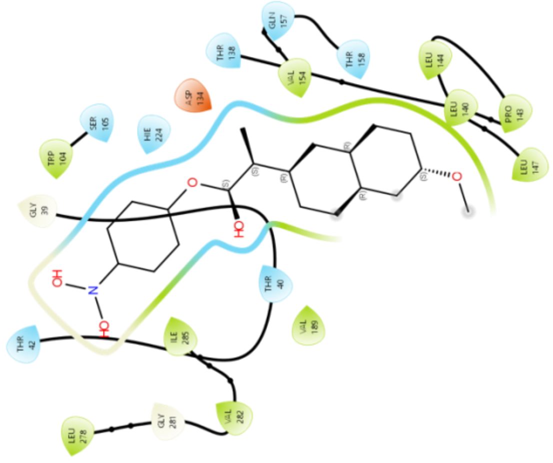

SG303 with rac-12

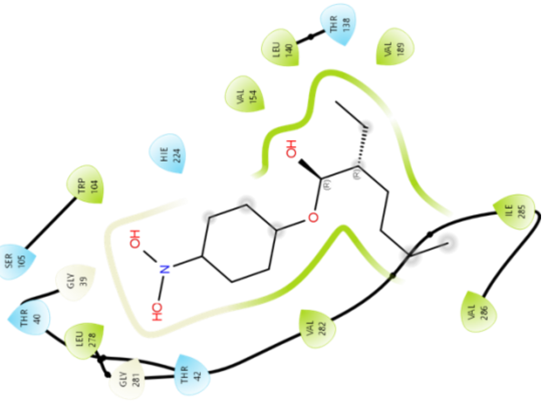

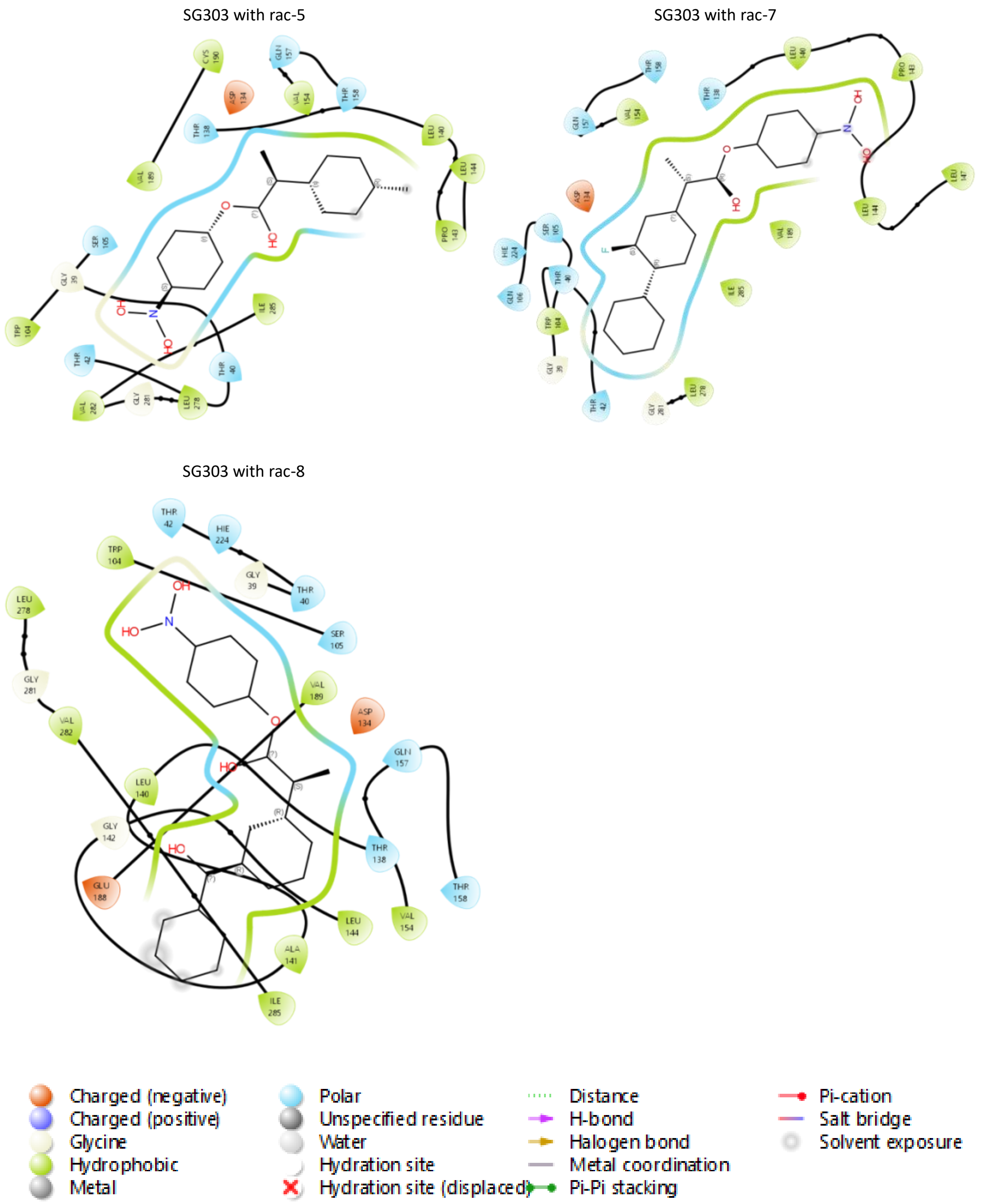

Figure S6 The 2D interaction maps around the substrate binding pocket in CalB. The complex structures were obtained by molecular docking followed by MD simulations. The graphs are generated by Maestro (Schrodinger Inc).

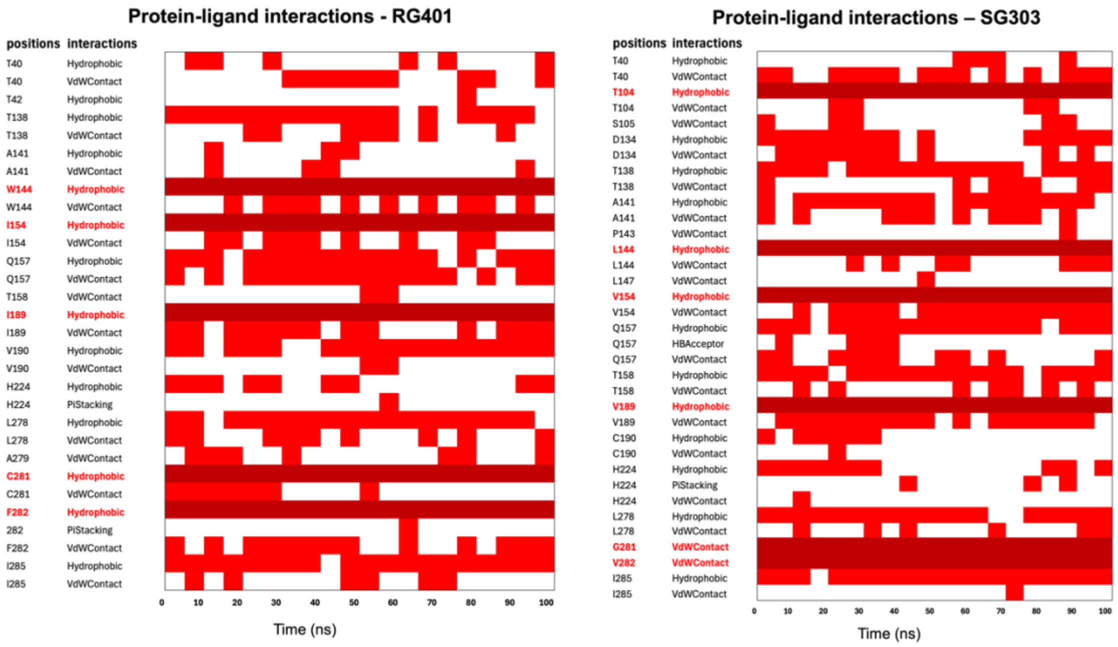

Figure S7 Detailed per-frame protein-substrate interaction fingerprints generated using ProLIF for mutant enzymes interacting with the ligand.

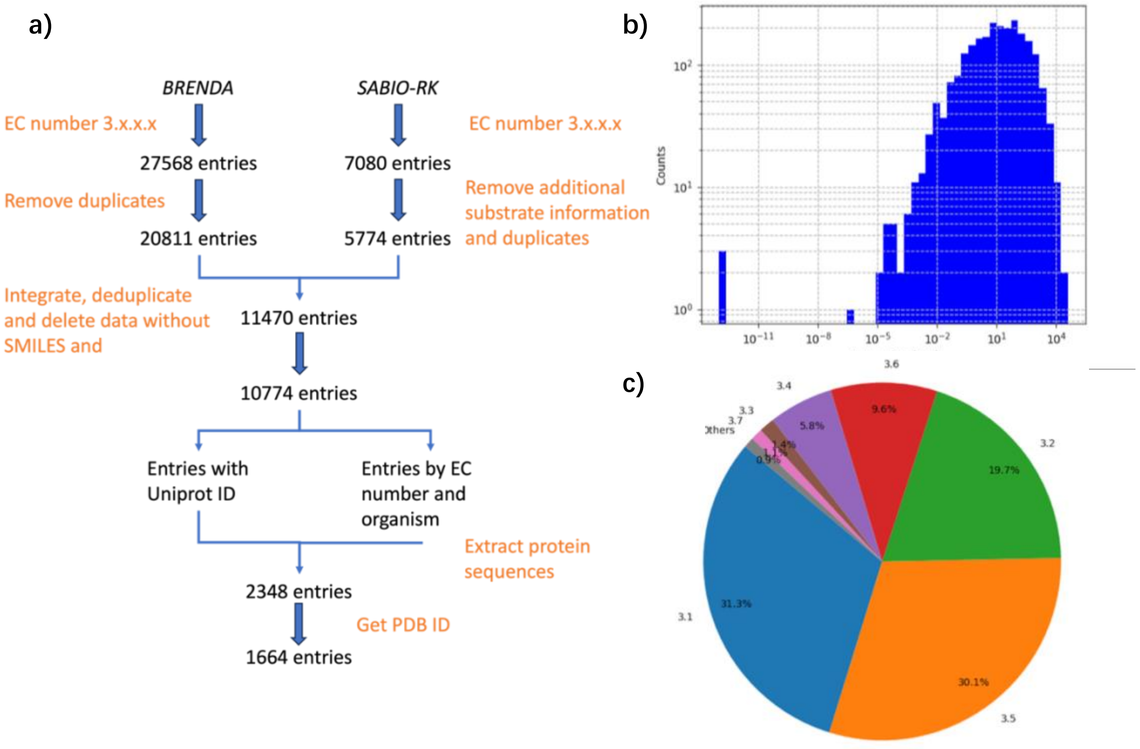

Figure S8 Data collecting and cleaning and the distribution of the Kcat dataset for enzymes involved in hydrolase activities (with EC number begin with “3”). **a).** The process involves removing duplicates and the irrelevant data, processing additional substrate information and categorizations, extracting protein sequence data, and ultimately obtaining PDB IDs. The scripts and codes are included in Github. **b).** The distribution of catalytic turnover numbers (kcat) across a dataset on a logarithmic scale. **c).** The categorization of enzymes based on their EC (Enzyme Commission) number classifications.

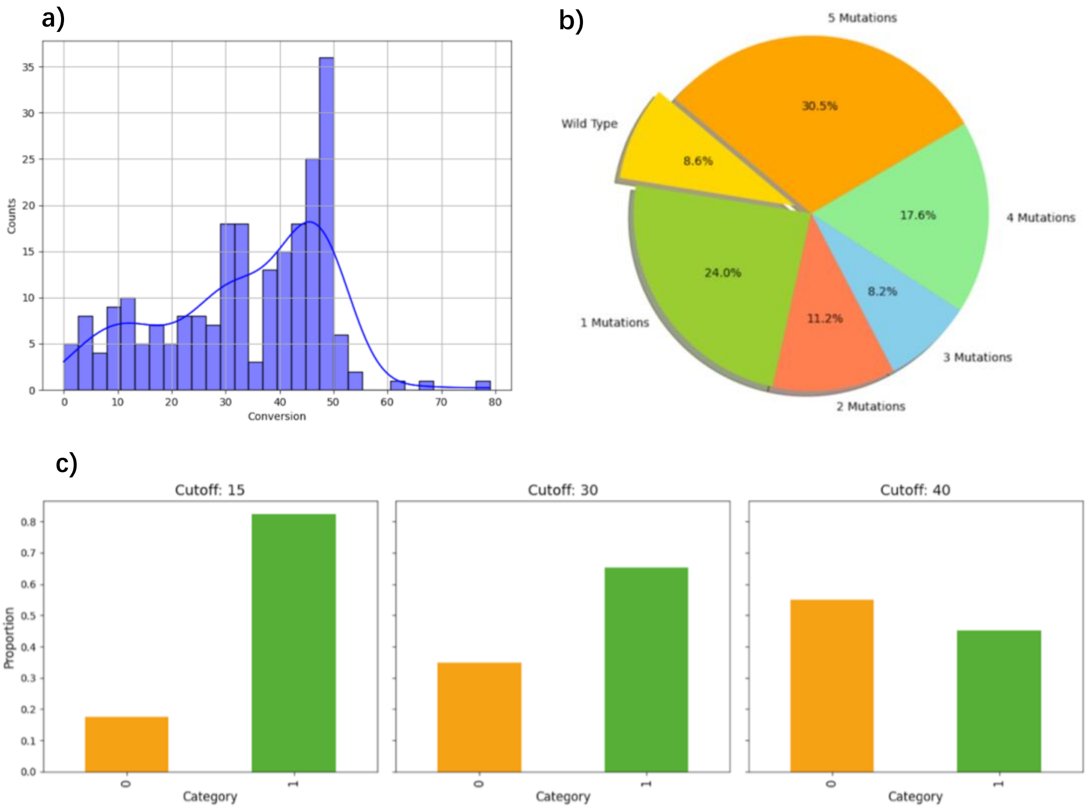

Figure S9 The detailed information of CalB conversion dataset. **a).** The distribution of conversion values in the CalB dataset. A kernel density estimate (KDE) is overlaid to indicate the general trend and concentration of data points. **b).** The proportions of wild-type and various mutation counts ranging from one to five mutations. **c).** The distributions of binary categorization of conversion values in the CalB dataset at cutoff thresholds of 15%, 30%, and 40%. “0” indicates lower than cutoff and “1” indicates equal or higher than cutoff.

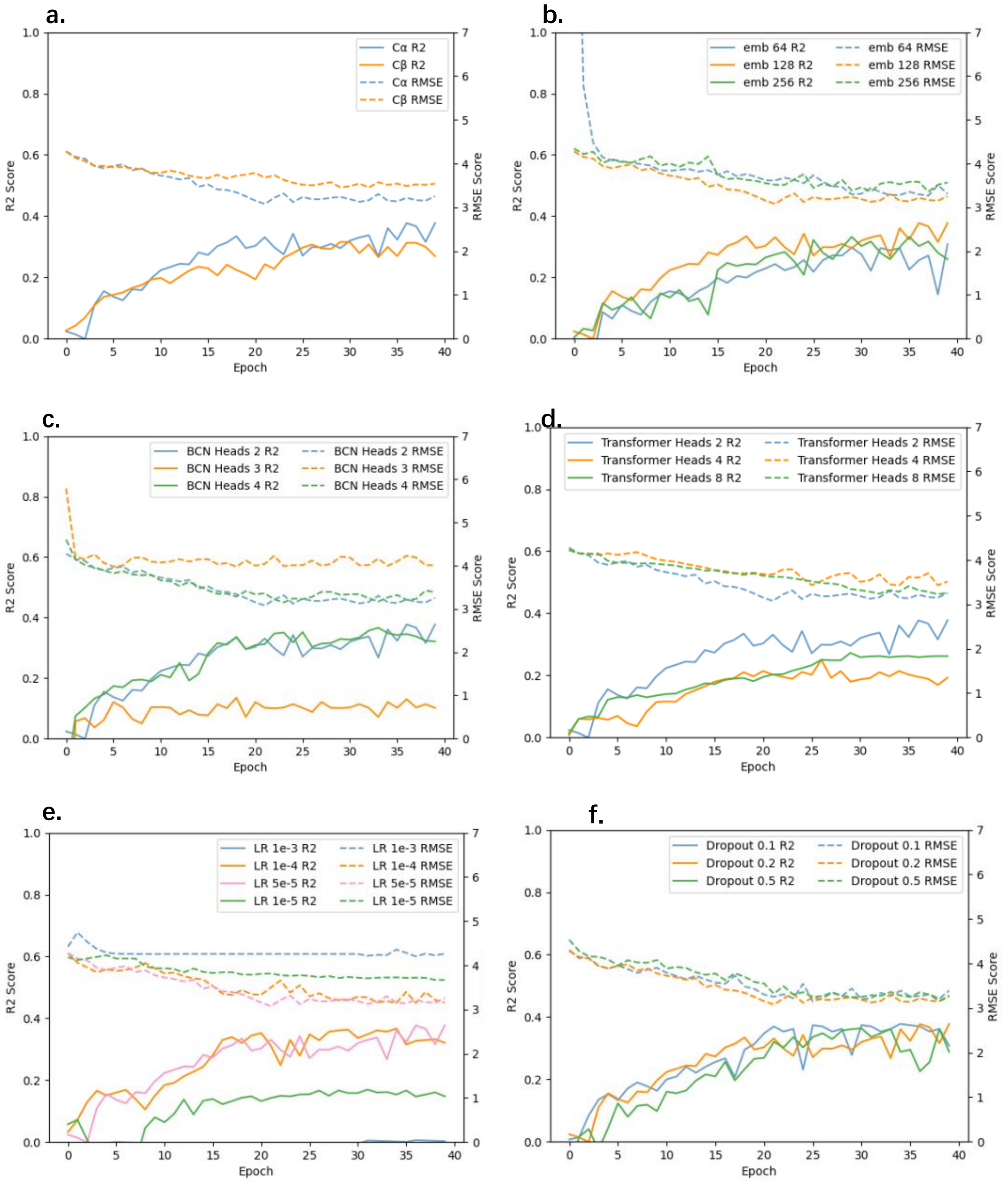

Figure S10 Learning curves of RMSE and R2 with various hyperparameters on the Kcat validation set. **a).** *Protein contact map*: The choices are Cα and Cβ*, which refer to the types of atoms used to define the contacts between a pair of amino acids in the protein structure. *For most amino acids, the Cβ atom is extracted except for glycine, where the Cα atom is used instead. **b).** *Hidden embedding*: The possible values are 64, 128, and 256. **c).** *attention heads in BCN models*: Available options are 2, 3, and 4. **d).** *attention heads in transformer-based models*: The choices include 2, 4, and 8. **e).** *Learning rate*: Possible values are 1e-3, 1e-4, 5e-5, and 1e-5. **f).** *Dropout*: The options are 0.1, 0.2, and 0.5.

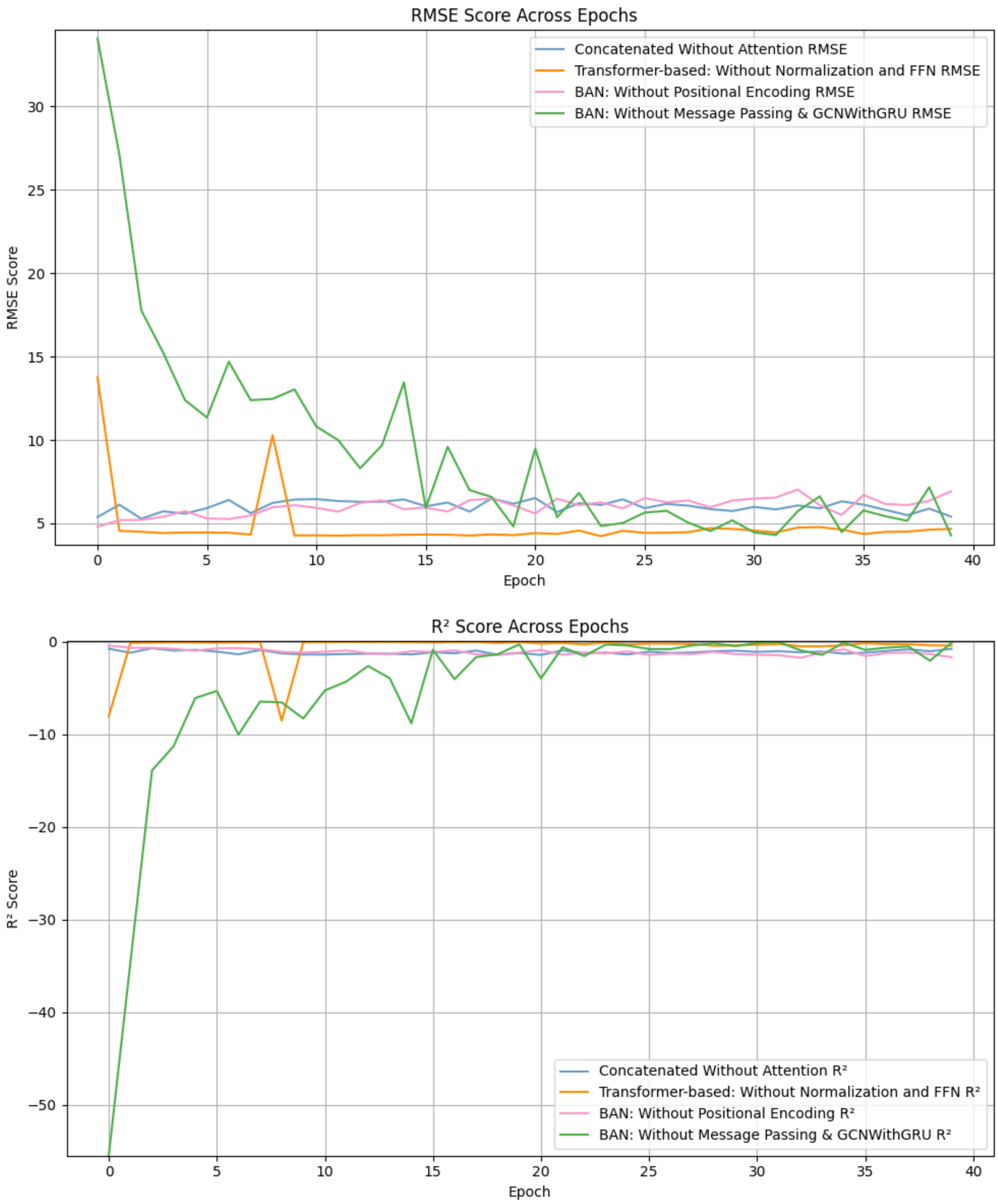

Figure S11 Learning curves of **a).** RMSE and **b).** R2 of ablation experiments on the Kcat dataset.
